# Supplementary material for: Deep learning-assisted comparative analysis of animal trajectories with DeepHL
Source: Nat Commun. 2020 Oct 20;11:5316. doi: 10.1038/s41467-020-19105-0 (PMC7576204; doi:10.1038/s41467-020-19105-0)
Supplement: Supplementary file 1 — Supplementary Information [file 41467_2020_19105_MOESM1_ESM.pdf]

Supplementary Information for

Deep Learning-assisted Comparative Analysis of Animal Trajectories with DeepHL

Takuya Maekawa, Kazuya Ohara, Yizhe Zhang, Matasaburo Fukutomi, Sakiko Matsumoto, Kentarou Matsumura, Hisashi Shidara, Shuhei J. Yamazaki, Ryusuke Fujisawa, Kaoru Ide, Naohisa Nagaya, Koji Yamazaki, Shinsuke Koike, Takahisa Miyatake, Koutarou D. Kimura, Hiroto Ogawa, Susumu Takahashi, Ken Yoda

Takuya Maekawa.
E-mail: maekawa@ist.osaka-u.ac.jp

Contents

User guide to DeepHL 2
Algorithm 3
Usage of Python-based Software 7
Significance of analysis of worms 8
Significance of analysis of red flour beetles 8
Application to the study of seabirds 8
Application to the study of crickets 9
Application to the study of bears 10
Evaluation with synthetic data 11
Effect of input features 11
Comparison with classic approaches 12
Animals 12

List of Figures

1 Supplementary Figure 1 . . . 14
2 Supplementary Figure 2 . . . 15
3 Supplementary Figure 3 . . . 16
4 Supplementary Figure 4 . . . 17
5 Supplementary Figure 5 . . . 18
6 Supplementary Figure 6 . . . 19
7 Supplementary Figure 7 . . . 20
8 Supplementary Figure 8 . . . 21

List of Tables

1 Supplementary Table 1 . . . 22
2 Supplementary Table 2 . . . 23
3 Supplementary Table 3 . . . 24
4 Supplementary Table 4 . . . 25
5 Supplementary Table 5 . . . 26

User guide to DeepHL

Quick start.

1. Access to DeepHL: DeepHL is accessible on the Internet through http://www-mmde.ist.osaka-u.ac.jp/~maekawa/deephl/. We have confirmed that DeepHL works with Google Chrome and Firefox. Using the links in the navigation bar at the top of this page, the user can go to various pages such as the DeepHL registration page or a page for managing comparative analysis jobs.

2. Creating an account: The user can go to the login page by clicking on the "Login" button in the navigation bar. If the user already has an account, he/she can log in with his/her user ID and password. Otherwise, the user clicks on the "Create account" button to go to a page for creating an account. The user inputs the required information such as a user ID and email address to create an account. An activation email is then sent to the registered email address. Activation is completed by visiting the URL given in the activation email. After completing the activation, the user can log in with his/her user ID and password from the login page.

3. User profile: The user can view his/her account information such as user ID and email address by clicking on the "Profile" button in the navigation bar at the top of each page.

4. Creating a job: DeepHL manages an instance of comparative analysis as a job. The user can browse a list of his/her registered jobs by clicking on the "Dashboard" button in the navigation bar (Supplementary Fig. 1a). The user can register a job by clicking on the "Add new job" button at the bottom of the dashboard page. Note that before registering a job, the user should prepare a zip file that includes the trajectory data files. The zip file should contain two folders: one containing the trajectory files belonging to one class and another containing the trajectory files belonging to the other class. The method of data preparation is detailed below. First, the user inputs a name and description of the job as text (see also Supplementary Table 4). When the trajectory data files include latitude/longitude information, the user specifies the indices of columns of the files that contain this information, which is used to show the analysis result (colored trajectories) on a map. When the user wants to use sensor data measured by the user and/or features calculated by the user as DNN inputs in addition to speed and relative angular speed, the user inputs the corresponding indices of columns in the trajectory data files that contain this information. Furthermore, the user can easily input simple commonly used primitive features into DeepHL-Net. If the user wants to use the distance from the initial point of each trajectory (straight-line and travel distances between the initial location and the coordinates at each time slice) as DNN input, the user selects "Use the distance from the start point as DNN input" checkbox. If the user wants to use the angle between the y-axis and the line segment connecting the start point and the coordinates at each time slice as a DNN input, the user selects "Use the angle from the start point as DNN input" checkbox. DeepHL has a function to automatically calculate these commonly used features and input them into the DNN. Finally, the user specifies the number of convolutional/LSTM layers of DeepHL-Net, number of nodes in each layer, and number of epochs for DNN training. (The batch size for training is 32.) Using the default values of these parameters is recommended. After registering the job, the user uploads the zip file including the trajectory data files at the dashboard page. Once the file upload is complete, the analysis starts automatically. The user can confirm the progress of the analysis from the "Information" buttons on the dashboard page. Detailed information about the jobs can be shown by clicking on the "Information" buttons as well.

5. Analysis result: After the analysis is complete, the user can browse the result by clicking on the "Analysis result" button. Supplementary Fig. 1b presents a screenshot of the result page. This page shows the result of the binary classification, i.e., classification accuracy, and a graph showing the transitions of loss and accuracy in the DNN training. When the classification accuracy is high, we can say that the trained DeepHL-Net is able to recognize the difference between trajectories belonging to the two different classes. From the graph showing the transitions of loss and accuracy, the user can determine whether DeepHL-Net was trained properly. If there is any difference between the trajectories belonging to the two classes, the loss gradually decreases and then eventually converges. If it does not converge, the user is recommended to increase the number of epochs and train again. This page further provides information about the scores of the convolutional/LSTM layers. Because layers in the network attempt to detect the difference between the two classes from different viewpoints (temporal scales), the user can acquire different types of knowledge from different layers. Therefore, DeepHL permits the user to select a layer to be used to color trajectories. The table shown in Supplementary Fig. 1c shows the score of each layer calculated using our scoring function, which is described in the **Methods** in detail. The column "score" of this table shows the scores of the layers. By clicking on each column header, the user can sort the layers by their scores in ascending/descending order. Therefore, the user can easily highlight trajectories using a high-score layer, i.e., a discriminator layer. The highlighting result for a layer can be shown by clicking on the "Highlight" button in the "Operation" column.

6. Highlighting result: Supplementary Fig. 1d is a screenshot of a page that shows trajectories highlighted by a selected layer. Two trajectories belonging to different classes are shown side-by-side, permitting the user to easily compare these two trajectories. Additionally, line graphs of the time-series of attention values for the trajectories are shown below the trajectories. The user can select a trajectory to show using a selection box. The items (trajectories) of the selection box

are sorted in descending order of scores. (The score of a trajectory is explained in detail in **Methods**.)
On this page, the user can also browse trajectories colored by the various handcrafted features listed in Supplementary Table 1 (Supplementary Fig. 1e). The user can select a feature to color the trajectories using a selection box. Furthermore, the user can color the trajectory using other sensor data or features handcrafted by the user and included in the uploaded trajectory data files. By comparing trajectories colored by attention values with trajectories colored by feature values, the user can visualize the relationship (correlation) between the attention and feature values. In addition to the above function, this page provides a table, such as the one shown in Supplementary Fig. 1f, that shows the correlation coefficient between the attention values of the selected layer and the values of each of handcrafted feature or sensor data. This table helps the user understand the meaning of the attention of the selected layer, in other words, it helps them understand which feature or sensor data strongly relates to the attention of the selected layer.

Data format. Here, we explain how the user prepares a zip file containing the trajectory files to be uploaded to DeepHL. First, the user creates a folder for each of the two classes to store trajectory files belonging to the class, i.e., the user creates two folders, for example, "male" and "female" folders. The folder name is used as a class name in DeepHL. Then, the user stores a CSV file prepared for each trajectory in its corresponding folder, e.g., a file containing a male trajectory is stored in the male folder. Finally, the user creates a zip file containing these folders. (For example, the user compresses the parent folder of these two folders in a zip file.)

We then explain the format of a CSV file containing a trajectory. The first column of the file corresponds to the timestamp (numeric), the second column corresponds to the X coordinate, and the third column corresponds to the Y coordinate. The first line of the file is the header, and each of the following lines describes the timestamp, X coordinate, and Y coordinate at each time slice. The time units of the timestamp are also used in DeepHL to calculate features such as speed.

Moreover, the user can add sensor data collected by the user as well as features calculated by the user in addition to the timestamp and coordinate values. The user can simply add the additional features/sensor data next to the Y coordinate value for each line. For example, if the user wants to include an ambient temperature, longitude, and latitude values for each time slice, each line should consist of the timestamp, X coordinate, Y coordinate, temperature value, longitude value, and latitude value in this order. (The longitude and latitude information can be used to show the trajectory on a map. Because of the Earth's curved surface, the X and Y coordinates of a two-dimensional flat surface converted from longitude and latitude values are sometimes used in trajectory analysis when target animals travel long distances. Therefore, this example includes X and Y coordinates as well as longitude and latitude values.) DeepHL is able to color trajectories using these sensor data and feature values, and it calculates the correlation coefficients between attention values and the features/sensor data. Furthermore, these sensor data and features can be easily input into DeepHL-Net.

Complementary information of DeepHL functions and additional functions. Here, we describe the above functions in detail as well as the additional functions provided by DeepHL.

- Classification accuracy: The classification accuracy shown in Supplementary Fig. 1b in the analysis result page is calculated based on the classification result when DeepHL-Net has been trained on 80% of the randomly selected trajectories and then tested using the remaining 20% of the trajectories. The "Accuracy" in Supplementary Fig. 1b is the percentage of the test instances (trajectories) correctly classified. In addition, the precision, recall and F-measure ($2 \cdot \frac{precision \cdot recall}{precision + recall}$) are calculated for each class.

- Classification result: Success or failure of the classification of each test trajectory is shown in the "Classification result of test trajectories" panel in the analysis result page. We recommend that the user ignore the misclassified trajectories when the user browses highlighted trajectories. In addition, DeepHL manages each trajectory using its index number, and the correspondence of the index number with the trajectory file name in the uploaded zip file is shown in "Trajectory information of CLASS_NAME" panel. (Here, "CLASS_NAME" is replaced by the name of the class.)

- Tile mode: In the highlighting result page, only one trajectory from each class is shown at the same time. The tile mode, which provides a holistic view of the highlighting, enables the user to visualize multiple highlighted trajectories at the same time, as shown in Fig. 4d in the main text. The user can enter tile mode by clicking on a button in the upper right portion of a highlighted trajectory in the highlighting result page.

Algorithm

Supplementary Fig. 2a shows an overview of our proposed method. We first preprocess input trajectory data and train DeepHL-Net for binary classification using the preprocessed training trajectories. Then, we classify each of preprocessed test trajectories and compute the classification accuracy. We also record the attention time-series of each layer in DeepHL-Net when each trajectory is fed into it. In addition, for each trajectory, we compute the score of that the trajectory belongs its class, which is used to screen trajectories by a user. Furthermore, as required by the user, our web-server based program provides various functions such as trajectory coloring. We explain the procedures of our method in detail.

Preprocessing. Let P be an input trajectory that consists of a sequence of two-dimensional positions with timestamps:

$$\begin{aligned} P &= [P_1, P_2, \cdots, P_T] \\ &= [(t_1, x_1, y_1), (t_2, x_2, y_2), \cdots, (t_T, x_T, y_T)] \end{aligned}$$

For the trajectories of animals whose absolute coordinates are meaningless, such as those of animals that freely move on an agar plate, the absolute position and rotation-invariant analysis is required. Therefore, we convert P into S, which is a sequence of speed and relative angular speed:

$$\begin{aligned} S &= [S_3, S_4, \cdots, S_T] \\ &= [(s_3, r_3), (s_4, r_4), \cdots, (s_T, r_T)], \end{aligned}$$

where s_i is speed at time i and described as

$$s_i = \frac{\mathrm{Dist}(P_i, P_{i-1})}{t_i - t_{i-1}},$$

where Dist() computes the Euclidean distance between two coordinates. In addition, r_i is relative angular speed computed based on the relative angle[1]. The relative angle (relative to the previous time slice) is the angular difference between the movement directions at times i and $i-1$. Note that because Vlachos et al.[1] assumes a uniform sampling interval, we compute relative angular speed by dividing the relative angle by the sampling interval. (The intervals of sensor data from sensor devices are not always uniform.) A movement vector at time i is defined as

$$V_i = P_i - P_{i-1}$$

Then, the relative angular speed at time i is defined as

$$r_i = \frac{\mathrm{sign}(V_i, V_{i-1}) \cdot \cos^{-1}\left(\frac{V_i \cdot V_{i-1}}{||V_i|| \cdot ||V_{i-1}||}\right)}{t_i - t_{i-1}},$$

where $\mathrm{sign}(V_i, V_{i-1})$ denotes the direction of the rotation (i.e., clockwise or counterclockwise) and is defined as

$$\mathrm{sign}(V_i, V_{i-1}) = \begin{cases} 1 & (([V_i \times V_{i-1}] \cdot [0\ 0\ 1]^T) > 0) \\ -1 & (otherwise) \end{cases}$$

Using the direction of the cross product between V_i and V_{i-1}, we can determine whether or not the animal turns towards the positive or negative z-axis. Finally, we iteratively normalize r_i to fix any oscillation around the π angle[1]. Specifically, we iterate the following computation n times:

$$r_i = r_i - \overline{r},$$

where $\overline{r}$ is the average of the relative angular speed values in a time-series of interest. In our implementation, $n = 10$.

Then, S, i.e., a sequence of two-dimensional vectors, is fed into DeepHL-Net. Note that in our system, the user can add sensor data or handcrafted features into the DNN inputs. When additional sensor data a and b have been added, the vector sequence becomes

$$S = [(s_3, r_3, a_3, b_3), (s_4, r_4, a_4, b_4), \cdots, (s_T, r_T, a_T, b_T)].$$

In addition, the user can easily include three additional kinds of primitive features into DeepHL-Net: the straight-line distance from time 1, travel distance from time 1, and angle difference from time 1. Because these features are automatically computed in our system, the user does not need to compute these features and include them in trajectory data files. The primitive features at time i are computed as follows:

$$\text{straight line distance}_i = \mathrm{Dist}(P_1, P_i),$$

$$\text{travel distance}_i = \sum_{n=2}^{i} \mathrm{Dist}(P_n, P_{n-1}),$$

$$\text{angle from init}_i = \mathrm{atan}(P_i - P_1).$$

As above, we compute S for each trajectory. Before the vector sequence is fed into DeepHL-Net, we standardize the elements of each dimension.

Network training. As described above, we obtain a multivariate vector sequence from each trajectory. Each sequence is associated with a ground truth class label, e.g., class A or class B. We train DeepHL-Net such that it classifies each trajectory into the appropriate class.

Network structure. The network structure of the proposed DNN model (DeepHL-Net) is described in the main text and **Methods**. DeepHL-Net consists of convolutional/LSTM layers and an output layer consisting of two nodes. Each node in the output layer outputs the class probability of its corresponding class. We employ the tanh function as the activation function of the nodes in the convolutional/LSTM layers. With this function, we can limit the output value of each node to a value between -1 and 1. We employ the softmax function as the activation function of nodes in the output layer. This function is used in classification tasks and makes the output classification probabilities sum to 1.

The convolutional neural network was originally proposed for image recognition, in which the convolutional layers were used to learn efficient filters for feature extraction from an image, i.e., two-dimensional data. Here, we employ convolutional layers to extract local features in time from a time-series of primitive features. Supplementary Fig. 2b shows an overview of the convolutional layers in our model. As mentioned in the **Methods** section, the input of the first convolutional layer is an $l_{MAX} \times N_f$ matrix, where l_{MAX} is the maximum length of the input trajectories, and N_f is the dimensionality of the time-series. In the first layer, we extract features from the input data by convolving values within an $F \times N_f$ filter, where different filter sizes F are used in different convolutional stacks. We use a stride of one sample in terms of the time axis and extract features using the sliding filter. Because we employ 16 filters in each layer, we obtain an $l_{MAX} \times 1$ output matrix for each filter. Note that, to maintain the length of the output to l_{MAX}, zero padding is applied to the inputs such that the output has the same length as the original input. In the following layers, an $F \times 1$ filter is used to obtain an $l_{MAX} \times 1$ output matrix.

LSTM is a type of recurrent neural network, which is an artificial neural network used for time-series analysis. With the LSTM layers, we abstract, i.e., extract features from, time-series data. LSTM has an RNN architecture with memory cells, and it can learn temporal relationships over a long time scale. A standard RNN fails to learn long-term dependencies because of the vanishing gradient problem [2]. LSTM solves this problem by employing memory cells that hold past information, updating the cell state using write, read, and forget operations with input, output, and forget gates [3]. Therefore, LSTM has been used to recognize time-series data, e.g., speech and human activity [4,5]. Supplementary Fig. 2c shows a diagram of an LSTM cell (node). The input at time t is $x_{t,j}$ and the output at time t at a node in the j-th layer is $z_{t,j}$. For LSTM cells in the first LSTM layer, $x_{t,1}$ corresponds to S_t. For LSTM cells in the other LSTM layers, $x_{t,j}$ corresponds to $z_{t,j-1}$, i.e., the output of each cell in the $(j-1)$-th layer. Therefore, simple features are extracted in the shallow LSTM layers and then abstracted concepts (features) are extracted in the deep LSTM layers. As Supplementary Fig. 2c shows, the outputs of the j-th LSTM layer at each time t are fed into the $(j+1)$-th LSTM layer at time t.

Training network. We trained the proposed binary classification DNN to minimize the cross-entropy between the distribution of the ground truth and a distribution estimated by the softmax output layer, employing backpropagation using Adam [6], which enables us to automatically adjust the learning rate. Note that the learning rate is a parameter that controls the rate at which parameters are updated in a neural network.

In this study, a node in the output layer of DeepHL-Net corresponds to one class. For example, when a trajectory belongs to class A, its ground truth is $[1.0,\ 0.0]$, where the first dimension corresponds to class A and the second dimension corresponds to class B. In backpropagation, the parameters of nodes in the layers of DeepHL-Net are iteratively updated to minimize the error (cross entropy) between output of a preprocessed trajectory and its ground truth. Here, because the initial parameters of each layer are randomly selected, the parameter values after training are different from layer to layer.

Recognition. Each test trajectory is fed into the trained DeepHL-Net to estimate a classification result of the trajectory. Based on the results, the accuracy, precision, recall, and F-measure, which appear on our web interface, are calculated. In addition, the attention values of all layers at each time slice, which are used to color the trajectory, are recorded.

Scoring layers. The task of the user is to find a discriminator layer for coloring trajectories from among the several layers included in the trained DeepHL-Net. To facilitate layer screening, we compute the score of each layer. For details about the score calculation, see the main text and **Methods**.

Ranking trajectories. After the user selects a discriminator layer, he/she can browse trajectories colored by the layer. To facilitate the screening of the trajectories to be shown to the user, we rank the trajectories belonging to each class. The scoring function used for the ranking is described in **Methods**.

Coloring trajectories. In Equation (1) in the main text, the softmax function ensures that all the attention values in a trajectory sum to 1. We translate each attention value using the color map shown in the right portion of Fig. 1a in the main text to color a trajectory so that the maximum attention value of all the trajectories corresponds to red and 0 corresponds to yellow. When an attention value at time t is a_t, a line segment connecting P_{t-1} and P_t is colored with a color obtained from $\mathbf{a}_t$.

Appendix: Artificial neural networks.

Basics of an artificial neural network. An artificial neural network typically consists of layers, as shown in Supplementary Fig. 2d. Each layer is made up of a number of interconnected nodes that contain activation functions. Input data are fed into the network via the input layer, which communicates with the hidden layers, where "processing" of the input data is performed. The hidden layers link to the output layer, where the processing result, e.g., the estimated class, is output. This simple

network is called a feedforward network. An example node shown in the right portion of Supplementary Fig. 2d has n inputs to the node, and an output of the node is calculated as follows:

$$z(x) = f\left(\sum_{i=1}^{n}(w_i x_i) + b\right),$$

where w_i is the the weight of the i-th input, which is multiplied by the value of the i-th input feature, and b is the bias. In addition, $f()$ is an activation function that is used to normalize the range of output values. We used the tanh function for nodes in the convolutional/LSTM layers and the softmax function for nodes in the output layer. The softmax function is used for classification tasks and it ensures the output values sum to 1. When an output layer has two nodes and an output of a node gives its corresponding class probability, e.g., the probability for class A, the sum of the two output class probabilities should be 1.

Training network. In this process, parameters in the network, i.e., w_i and b in each node, are estimated based on training data. Training data consist of a set of pairs of an input vector and output vector, i.e., the ground truth. In the case of binary classification, an output vector corresponding to the first class, for example, is [1, 0]. The backpropagation algorithm[7] is used to estimate the network parameters that give small prediction errors for the training data. The backpropagation algorithm can be regarded as *learning from mistakes*. The algorithm first randomly initializes the network parameters and then updates the parameters in an iterative process. In each iteration, a selected training input vector is fed into the network and its output vector is computed. When the computed output is compared with the ground truth and is found to be wrong, i.e., different from the desired output, the algorithm updates the network parameters to correct the mistake. The difference between the calculated and desired outputs, i.e., the error, is propagated from the output layer to the input layer and then the algorithm calculates the partial derivative of the propagated error with respect to each parameter to reduce the error. For more details about the backpropagation algorithm, refer to Ref.[7].

Recurrent neural network (RNN). RNNs are usually used to recognize sequential data such as text, speech, and video. The principal feature of RNNs is their ability to utilize previous information for the present task, e.g., using previous video frames to understand the present frame. The left portion of Supplementary Fig. 2e shows an example of the architecture of a simple RNN. Here, the recurrent node has a connection from its output back to its input. This is called the feedback connection and it enables the output of the present time to be calculated from both an input of the present time and output of the previous time. The network is also trained by backpropagation. The backpropagation with the recurrent connection is simply performed by unfolding the network as shown in the right portion of Supplementary Fig. 2e. The example network is unfolded into a network with a depth of t. After unfolding, the network can be trained in almost the same way as a feedforward network with backpropagation. The algorithm for recurrent nets is called backpropagation through time (BPTT). For more details, refer to Ref.[8].

Long short-term memory (LSTM). LSTM is a type of RNN model where the recurrent nodes are replaced by LSTM cells consisting of memory cells. The aim of LSTM is to solve a problem of RNNs called the vanishing gradient problem[3], where errors (the difference between the desired and calculated outputs) vanish through the deep backpropagation. The structure of an LSTM cell is shown in Supplementary Fig. 2c. The cell stores an internal state called a memory cell C_t, allowing the RNN to write information to the cell, read information from the cell, or forget the information in the cell. These operations are performed by the forget gate f_t, input gate i_t, and output gate o_t, and these gates and the cell state are mathematically described as follows.

$$\begin{aligned}
f_t &= \sigma(W_f \cdot [z_{t-1}, x_t] + b_f),\\
i_t &= \sigma(W_i \cdot [z_{t-1}, x_t] + b_i),\\
C_t &= f_t * C_{t-1} + i_t * \tilde{C}_t,\\
\tilde{C}_t &= \tanh(W_c \cdot [z_{t-1}, x_t] + b_C),\\
o_t &= \sigma(W_o \cdot [z_{t-1}, x_t] + b_o),\\
z_t &= o_t * \tanh(C_t),
\end{aligned}$$

where z_{t-1} is an output vector at time $t-1$, x_t is an input vector at time t, and $\sigma()$ is the sigmoid function, which is used to limit the input value to a value between 0 and 1 as follows.

$$\sigma(x) = \frac{1}{1+e^{-x}}$$

Moreover, f_t is the output of the forget gate at time t, and W_f and b_f are the weights and bias of the gate (node), respectively. Based on the present input x_t and previous output z_{t-1}, the forget gate determines whether or not it will forget previous cell state C_{t-1}. Output f_t is multiplied by cell state C_{t-1} when C_t is computed to determine whether the cell forgets everything or keeps the information. Output i_t is an output of the input gate at time t, and W_i and b_i are the weights and bias of that gate, respectively. The input gate computes the update for the cell based on x_t and z_{t-1}. Output i_t determines the effect of $\tilde{C}_t$ on C_t. Here, $\tilde{C}_t$ is a candidate of C_t calculated based on z_{t-1} and x_t. Parameters W_c and b_C are the weights and bias of the node that calculates $\tilde{C}_t$. Output o_t is an output of the input gate at time t, and W_o and b_o are the weights and bias of the gate, respectively. The output z_t of the LSTM cell at time t is determined by o_t and C_t.

Usage of Python-based Software

For biologists who can set up deep learning environments, we also provide the Python-based software for DeepHL, which runs on an Ubuntu computer with a GPU board (DOI:10.5281/zenodo.4023931). We tested the operation of our software on a desktop computer with the following specifications: Intel Core i7-6700K CPU, 32 GB memory, Nvidia GeForce GTX Titan X GPU, and the Ubuntu 14.04.3 LTS operating system. In addition, the following software was installed: Anaconda v4.5.11, CUDA v8.0.0, CuDNN v7.0.5, Python v3.6.6, Tensorflow (gpu): v1.4.1, and Keras (gpu): v2.0.8.

It takes about half hour to install the above software. Our software contains the following five main files:

- preprocessing.py: for preprocessing trajectory data.
- train.py: for training DeepHL-Net on training data.
- test.py: for computing the test accuracy of the trained DeepHL-Net and attention values of the trajectories.
- activation_node.py: for computing the scores of layers and trajectories.
- plot_trajectory.py: for plotting the colored trajectories.

Because our programs are written in Python, it takes little time to install the software. The user can configure several parameters such as the dropout rate and network size. For more detail, refer to the readme document associated with the Python files. Supplementary Fig. 2f and 2g shows screenshots of the user interface of plot_trajectory.py. This program visualizes the colored trajectories belonging to different classes side-by-side, as shown in Supplementary Fig. 2f, as well as the time-series of attention values and feature values, as shown in Supplementary Fig. 2g. The user can change the trajectories to be shown by scrolling the mouse wheel. The user can also use the cursor keys to change the layer used for coloring. The user can switch to a mode that plots the time-series data using the Ctrl key and change the features to be plotted using the space and shift keys. For more details about the software, refer to the readme document associated with the software.

We also provide an example data set of worm data with our software. Here, we present the commands for running our software using the example data set. It takes about ten hours to complete the demo.

1. Preprocessing data of the naive worms:

```
python preprocessing.py -i 0 -s './dataset/naive' -d './wormdata/naive' -a 11101
```

Here, "./wormdata/naive" is the directory that contains the data of the naive worms. The preprocessed data are stored in "./dataset/naive". For more details, refer to the readme document.

2. Preprocessing data of the worms with learning:

```
python preprocessing.py -i 0 -s './dataset/preexp' -d './wormdata/preexp' -a 11101
```

3. Training DeepHL-Net on the preprocessed data:

```
python train.py -d './dataset' -r './result' -w './model' -n naive -m preexp -b True -e 0 -x 100 -u 128 -l 4
```

4. Testing the trained model and computing attention values:

```
python test.py -d './dataset' -r './result' -w './model' -n naive -m preexp -b True
```

5. Computing the scores of layers and the score of the trajectories:

```
python activation_node.py -d './dataset' -r './result' -w './model' -n naive -m preexp -b True
```

The results of classification are stored in "result.txt." Because our algorithm relies on deep learning, the reproducibility is not guaranteed. The scores of the layers are stored in "attention_score.csv," the histograms of the attention values are stored in the "hist" directory, and the correlation coefficients with features are stored in "correlation.csv."

6. Visualizing trajectories:

```
python plot_trajectory.py -d './dataset' -r './result' -w './model' -n naive -m preexp -b True
```

Significance of analysis of worms

C. elegans is regarded as a model animal for understanding the neural/molecular mechanisms controlling behavior. For machine-learning based behavioral analysis of *C. elegans*, several studies on feature extraction using decision-tree analysis or the analysis of their posture sequence have been reported[9–11]. In this study, we aimed to extract the features of odor-avoidance behavior modulated by learning. In learning, prior experience causes long-lasting neural activity, which results in changes in their behavior. Thus, identifying the changes in behavior caused by learning should allow us to estimate the neuron(s) in which the traces of experience (i.e., memory) exists. However, it is difficult to identify the characteristic behavioral changes from a continuously changing series of postures during movement over a long period of time.

Here, we focus on the repulsive odor learning in *C. elegans*. Kimura et al. found that preexposure of the worms to a repulsive odor 2-nonanone enhances odor avoidance behavior[12]: the preexposed worms move further away from the odor source more efficiently than naive worms. Interestingly, the average speed of the worms with and without learning were not significantly different, suggesting that the preexposed worms avoid the odor more efficiently. To determine all behavioral features characteristic of the repulsive odor learning, Yamazaki et al. performed a machine learning analysis using a decision tree[13], although because of the limitation of the method, they were not able to reveal the dynamic features of the time-series data.

Significance of analysis of red flour beetles

Tonic immobility (TI), sometime called as “thanatosis” or “death-feigning,” is an antipredator behavior of many animals[14–16]. Miyatake et al. performed a two-way artificial selection for the duration of TI, and established the strains with short (S-strain) and long (L-strain) duration of TI in the red flour beetle, *Tribolium castaneum*[17]. Miyatake et al. also revealed that beetles of the S-strain showed significantly higher levels of brain dopamine expression and a higher locomotor activity than those of the L-strain[18]. *T. castaneum* is an insect model species for which all the genomes are already known[19]. Hence, if the mechanisms controlling TI and locomotor moving traits can be clarified, we can investigate the molecular base of TI and movement. This might reveal the relationship between the nature of walking trails and Parkinson’s disease, which is affected by dopamine expression levels and influences walking patterns. However, nobody has yet compared the walking trails of S- and L-strain beetles.

Application to the study of seabirds

We provide an example of the analysis of streaked shearwaters (*Calonectris leucomelas*) to show the utility of DeepHL for the comparison of the behavioral patterns between males and females in large-scale movements (several hundreds of kilometers). Sexual differences in morphology, size, and behavior are common phenomena in the animal kingdom[20]. Previous studies on sexual differences have helped us to understand natural selection and the sexual selection of animals, but the large-scale movement of animals makes it difficult to detect the differences in behavior between sexes. Recently, animal-borne sensing approaches allow ecologists to record information about animal behavior such as GPS location or acceleration[21,22] and to relate sexual differences to environmental conditions[23]. However, comparison methods have been limited to the calculation of simple statistics (e.g., foraging trip duration and daily distance covered) that are based on expert hypotheses and prior knowledge. This approach might fail to fully utilize big time-series data that contain useful information for the comparison of behavioral patterns between sexes; therefore, advanced methods are required to go beyond simple statistical comparisons.

Streaked shearwater exhibits sexual size dimorphism, the males having a larger body mass and wing loading[24,25]. This might make each sex adapt to their environment by performing different behavior because body size and wing loading affect tolerance to wind[26,27], flight speed[28], and the energy expended for flight[29,30]. Such a difference in micro-movement properties might be organized into ecological differences between the sexes observed in foraging trip duration and distance travelled[25]. However, limited comparisons have failed to discover differences in movements at a number of different spatial scales, especially at the small scales.

We analyzed the trajectories of male and female streaked shearwaters living on Awashima Island (38°28’N, 139°14’E; Niigata, Japan) using DeepHL. We use 424 trajectories collected with a sampling interval of about one minute from 148 birds (70 males and 78 females) using GPS loggers (GiPSy-2, $37 \times 16 \times 4$ mm or GiPSy-4, $37 \times 19 \times 6$ mm; TechnoSmArt, Roma, Italy) attached to the back feathers of the birds (Supplementary Table 2). Each trajectory corresponds to a foraging trip, starting with the departure from the colony and ending with arrival back at the colony (or the GPS logger running out of the battery). Note that a foraging trip is defined as the time a bird spends beyond a 3-km buffer zone around the colony. Because few error measurements are included, we feed the preprocessed trajectories into DeepHL. To remove the error measurements, we first compute the movement speed for each data point and then simply discard data points with speeds faster than 80 km/h, which is the upper bound for birds.

We computed movement speed and relative angular speed from X and Y coordinates on a two-dimensional flat surface converted from longitude and latitude using R’s rgdal package (https://cran.r-project.org/web/packages/rgdal/index.html, accessed on 26 June 2018). In addition to movement speed and relative angular speed, the longitude, latitude, distances from the initial position, and angle from the initial position were fed into DeepHL-Net because the absolute coordinates of specific places such as colonies and feeding sites can affect the behavior of the seabirds. The accuracy of the binary classification between males and females was 65.7%. Supplementary Fig. 3a shows typical examples of trajectories highlighted using the attention of a discriminator layer. The score of the discriminor layer was the highest of all layers. As shown in the figure,

the layer pays attention to the male trajectory when the male bird travels directly away from the coastline. In contrast, the female trajectory is highlighted when the female bird stays close to the coastline. (We found similar patterns in other trajectories. Supplementary Fig. 3e shows a function of DeepHL enabling to compare multiple trajectories at a glance.) In addition, Supplementary Fig. 3b shows the angle from the initial position and attention values used for highlighting trajectories in Supplementary Fig. 3a, indicating increases in the attention values when the male bird travels directly away from the coastline. In fact, DeepHL indicated that the attention values of the layer highly correlate with the angle from the initial position (highest; see Supplementary Table 3). As for the female trajectories, the attention values are highly correlated with the longitude values (second highest). Because the coastline runs north–south, the distance between the coastline and a position relates to the longitude of the position. Supplementary Fig. 3c shows distributions of the GPS locations of all the male and female birds, indicating that GPS measurements of the female birds are closer to the coastline than those of the male birds.

We further analyzed the sexual difference in the distance from the coastline and found that the distance from the coastline for the female birds is significantly closer than that for the male bird (Supplementary Fig. 3d). Because the wind direction at an observation point closest to the island (38°75'N, 139°25'E) is southwest, which is computed from CoastWatch WCRN data (Wind, Metop-A ASCAT, 0.25°; https://coastwatch.pfeg.noaa.gov/erddap/griddap/erdQAwindmday.html, accessed on 26 June 2018) during the data collection periods and the average wind direction was 55.4°, it is believed that the female seabirds moved to the lee shore. (Note that the wind direction is defined as the direction from which the wind is blowing measured clockwise from north.) This is because the body size, which relates to the ability to fly against the wind, of the female birds is smaller than that of the male birds[23,28,31]. Although the sexual difference of the streaked shearwaters according to the distance from the coastline has not yet been investigated, we were able to obtain the above information from the layer that focuses on a specific geographical region and travel direction. Note that Sakuma et al. proposed a method to find frequent patterns for male or female seabirds using classic machine learning and investigated the sexual difference in the distance from the coastline[32]. However, the handcrafted feature, i.e., the distance from the coastline, used in this study was designed based on a finding of DeepHL. As above, the features discovered by DeepHL can also be utilized in classic machine learning methods.

Application to the study of crickets

Escape behavior is essential for animals' survival. The performance of the escape, including its moving trajectory, is flexibly altered by the surrounding context[33]. Here, we employed DeepHL to analyze the context-dependent modulation of the escape behavior in field crickets, *Gryllus bimaculatus*. Fukutomi et al. revealed that an acoustic stimulus at high frequency (>10 kHz) preceding an air puff alters crickets' moving direction in wind-elicited escape behavior[34,35], suggesting that the crickets recognize the high-frequency sound as the echolocation signal of bats and change their behaviors in the presence of predators. However, it is unclear what motion in the response modulates the escape trajectory because this acoustic stimulus has no impact on either the maximum or average value of locomotion parameters such as moving speed. Unfortunately, differences in the time-series of the locomotor parameters have not been considered due to a lack of analytical methods.

Here, we adopted DeepHL to compare two groups of escape movement: with-tone and without-tone. Based on two-dimensional coordinates and body-axis angle obtained with a spherical treadmill (Supplementary Fig. 4a; tracking software: Tracktaro, v. 1.0.4), the translational and rotational speeds were measured with a 200 Hz sampling rate. The cricket's walking activity was recorded using two optical mice that detect the rotation of an air-lifted trackball and output the X/Y position and body-axis angle in the virtual space. The air-puff and sound stimuli were delivered from nozzles and speakers located on the same side lateral to the cricket. For the with-tone protocol, a 10-kHz tone sound of 1-s duration started 800 ms before an air-puff for 200 ms. For the without-tone protocol, the air-puff was delivered without any preceding sound stimulus. In this experiment, we analyzed the short-term trajectories, defined as the "initial response" according to the following criteria: the initial segment of the response is the point at which the translational velocity exceeds 0.01 m/s or the angular speed exceeds 0.01 deg/ms, and the end of the segment is the point at which translational velocity and angular speed fall below 0.01 m/s and 0.01 deg/ms, respectively (see also Ref.[35]).

DeepHL-Net was fed with the primitive features, i.e., translational speed, angular speed, the distances from the initial position, and the angle from the initial position, as well as the measured rotational speed. The trained DeepHL-Net showed a classification accuracy of 73.6% for the with- and without-tone groups. We used a discriminator layer with the second highest score. Supplementary Fig. 4b shows typical trajectories colored by the attention values of a discriminator layer. The attention values increased near the middle of the trajectories. We then focus on the time-series of the attention value and rotational speed, which are highly correlated to each other (Supplementary Table 3). The attention value increases coincided with a peak in rotational speed, and then maintained high values in the without-tone trajectory. In contrast, the attention value increased in the with-tone trajectory just after the short-lasting peak of the rotational speed. Therefore, DeepHL seems to detect the difference in the peaks of the rotational speed for the without-tone and with-tone classes. The high rotational speed was sustained in the without-tone trajectory, which means that the crickets exhibited longer and larger turning movements in the without-tone group. In contrast, the rotational speed transiently elevated and peaked earlier in the with-tone group ($t = 35$ for with-tone; $t = 125$ for without-tone; Supplementary Fig. 4c). Averaged data for each individual in the without-tone group showed a delayed peak and long-lasting rise in rotational speed (Supplementary Fig. 4d,e). Therefore, we computed the delay to peak, which is the time delay from behavioral onset to time of the peak of angular speed, and the half width, which is the duration over which the normalized angular speed is higher than half (0.5) of the peak value in each trajectory. These two values were computed for each trajectory and then compared for the with-tone and without-tone groups (Supplementary Fig.

4f,g). We confirmed the significant differences in the delay to peak and half width using GLMM with Gaussian distributions (delay to peak: $t = 2.523$; $df = 14.493$; $p = .0239$, effect size(r^2) = 0.180, half width: $t = 3.124$; df = 15.254; $p = .00685$, effect size(r^2) = 0.088). All p-values are two sided. Individual factors were treated as random effects. The number of data points for the with-tone group is 206 and that for the without-tone group is 240. We used the lmerTest package (v. 2.0–36) of R (v. 3.4.3) for the analysis.

Thus, DeepHL analysis elucidated details of the temporal dynamics in the auditory-modulated escape behaviors that were not revealed by conventional analysis. The crickets exhibit slow and large turns in response to a lateral puff, whereas crickets hearing a high-frequency sound preceding the puff turned more quickly. Understanding the temporal dynamics of motor output is useful for exploring the neural mechanisms of the context-dependent modulation of escape behavior. In our cricket study, for example, the auditory inputs of the preceding sound could affect the activities of neurons that control the rotational speed in the turning locomotion.

Application to the study of bears

The major method of behavioral research for large terrestrial mammals has historically been conducted by direct observations[36]. However, direct observation is not always possible nor desirable. It is often unrealistic, biased, or difficult to perform under field conditions, such as in studies of forest species, nocturnal animals, and species that are highly cautious. After the 1960s, pioneering studies on these animals have been conducted based on very high frequency (VHF) radio telemetry data and obtained some fragmented behavioral information, but the use of GPS telemetry data provided detailed information both temporally and spatially up until the 2000s. However, because there were no differences between the information obtained using traditional VHF radio telemetry and that acquired by using GPS telemetry, e.g., calculation of the home range size or habitat selection, there has been some criticism about the fact that no new ecological knowledge has been obtained using GPS telemetry of large terrestrial mammal[37]. One of the reasons for this is that there are no effective methods for analyzing big behavioral data obtained by GPS telemetry.

The Asian black bear (*Ursus thibetanus*) is an ideal species for investigating the role of some factors on proposed behavior patterns. It is known that their body size sexual dimorphism (male > female) and the home range size of males is also larger than that of females even though they are a solitary mammal species. During late summer to autumn, bears need to fatten themselves before winter denning (hyperphagia period). However, the biomass of their dominant foraging item, hard mast, experiences large annual fluctuations[38]. Several studies have shown that the food scarcity that results from the autumn masting yield causes bears to change their feeding habits, expand their home-range size, and increase long-distance movement[39–41]. This also increases the chances of intrusion on human settlements and human-bear conflicts. Additionally, in poor mast years, previous studies have suggested that, in autumn, females have a greater tendency to move into human settlements than males[42]. Thus, clarifying the sexual differences in behavior patterns may help the coexistence between humans and bears.

The study was conducted in the Ashio-Nikko Mountains, in the central part of Honshu Island, Japan (approximately 460 km^2; 36°54'–36°80'E, 139°22'–139°49'N). Bear capture and handling methods are described in detail later and were performed in accordance with the guidelines for animal research established by the Mammal Society of Japan. To avoid potential bias due to capture, data collected in the 48 hours following release from capture were excluded.

We analyzed the trajectories of male and female bears using DeepHL. The number of trajectories is 552 and each trajectory corresponds to one week of data (Supplementary Table 2). Note that we discarded trajectories with small numbers of GPS measurements (<30). In addition to movement speed and relative angular speed, the longitude, latitude, distances from the initial position, and angle from the initial position were fed into DeepHL-Net. The classification accuracy for the binary classification between male and female was 59.8%. We focused on the discriminator layer that had the highest score of all layers. Supplementary Fig. 5a shows typical examples of trajectories highlighted using the attention of the layer. As shown in the figure, the layer pays attention to a segment of the male trajectory only. Our investigation of the trajectories of the male bears revealed that the layer pays attention to male trajectories when a male bear has traveled long distance before/after remaining in one place for a long time. (Supplementary Fig. 5c shows multiple trajectories of male bears provided by DeepHL.) In contrast, this layer does not pay attention to the female trajectories even when the female bear remains in one place, as shown in Supplementary Fig. 5a. In addition, Supplementary Fig. 5b shows the travel distance from the initial position and attention values used for highlighting trajectories in Supplementary Fig. 5a, indicating that the layer outputs large attention values when the travel distance suddenly increases and then remains high, i.e., when the bear stays in one place before/after movements over long distances. From the above results, it is assumed that the male bears change their locations more frequently than the female bears.

To investigate the sexual differences in the number of locations at which a bear stays for a long time, we performed cluster analysis using the DBSCAN algorithm[43]. The DBSCAN algorithm does not require the number of clusters as an input parameter, unlike the k-means algorithm[44], which is the most common clustering algorithm. The DBSCAN algorithm is suitable for extracting the locations at which a bear remains for a long time because the algorithm clusters data points according to the distances between them and detects outlying data points. To extract the number of locations from GPS data points collected over a fixed period, a trajectory corresponding to the data points of 50-day data was fed into the DBSCAN algorithm. (A total of 44 and 79 50-day trajectories from the male and female bears were used, respectively. The DBSCAN parameters of eps and minPts were 200 m and 6, respectively. Four male bears that have trajectories shorter than 50 days were discarded in this analysis.) As a result, 5.6 and 3.8 clusters were extracted on average from the male and female data,

respectively. Supplementary Fig. 5d shows the distributions of the number of clusters. The number of clusters of the male bears is significantly larger than that of the female bears. In addition, the numbers of average cluster members, i.e., data points, for the male and female bears are 25.8 and 52.4, respectively, indicating that the female bears stayed in one place twice as long as the male bears.

These results show that the males combined long distance movements with short rest periods at many clusters, and the females stayed in a limited number of clusters for a long time. Previous studies have suggested that bears generally make intensive use of a small area (or "core area": cluster in this study) over most of the year[45]. However, nobody knows whether there are sexual differences in the number of clusters used or the time spent at each cluster. Hence, they may be influenced by sexual differences in behavior patterns. In summer, the males moved frequently to search for females as breeding partners because summer is breeding season. In contrast, the females do not move frequently because they may prioritize food resources, which are limited during summer. Additionally, because the males are dispersed farther from their natal areas than the females[46], they may have more knowledge about a wide range of the habitat. This travel distance results indicates that the males may select the clusters they stop at randomly based on their memory. In contrast, because the females do not disperse much from their natal areas[47], they may have the home-court advantage in their habitat. Thus, the females do not move without reason and may stay in a limited number of clusters until they run out of food resources. These results indicated a sexual difference in the bears' behavior: the males have a flexible habitat use and the females have a conservative habitat use.

Evaluation with synthetic data

Data set. Here, we evaluate our method using synthetic trajectory data. We assumed two animal classes; class A and class B, and generated one hundred synthetic trajectories for each class. Animals belonging to class A have two latent states and animals belonging to class B have three latent states. When an animal is in a latent state, it moves according to movement speed and angular speed sampled from normal distributions prepared for the state, as listed in Supplementary Fig. 6a. The movement speed and angular speed were sampled for each time step (1 s). As shown in Supplementary Fig. 6a, the third latent state of class B generates the characteristic trajectory segments of the class. In addition, to simulate movement direction changes, the signs (plus/minus) of the means of the normal distributions of the angular speed were randomly switched with a probability of 10% at each time step. A state transition occurs every 10 s, and the duration of each trajectory is 100 s. An animal belonging to class B is in the third latent state three times (30 s in total) for each trajectory. Supplementary Fig. 6b shows the time-series of speed of an example trajectory of an animal belonging to class B. Segments with speeds of around 15 m/s correspond to the third latent state.

Evaluation methodology. The two hundred trajectories were fed into the DeepHL method. We then evaluated the detection performance of the third state using a discriminator layer with the highest score in the convolutional stacks. That is, we assumed binary classification where the first class corresponds to data points (locations) generated by the third latent state and the second class corresponds to data points generated by the first or second latent states. Supplementary Fig. 6c shows a trajectory corresponding to Supplementary Fig. 6b colored by its attention values. Supplementary Fig. 6b also shows the time-series of the attention values. Because the discriminator layer outputs attention values larger than 0.012 almost exclusively for high-attention segments of class B trajectories, as shown in Supplementary Fig. 6b, we assume that data points with attention values larger than 0.012 are classified into the first class by the discriminator layer. Otherwise, data points are assumed to be classified into the second class. We then computed the classification performance of the estimates.

Results. As shown in Supplementary Fig. 6b,c, the discriminator layer precisely detects segments corresponding to the third latent state. (Red segments show characteristic segments of class B.) Supplementary Fig. 6d shows the classification performance. The classification accuracy was about 70%. We confirmed that the boundaries between the segments of the third latent state and the first/second latent states detected by the attention of the layer are ambiguous.

Effect of input features

We extract primitive features, i.e., the distances from the initial position and angle from the initial position, in addition to speed and relative angular speed, from each trajectory. Here, we investigate the effect of the additional features (distances from the initial position and angle from the initial position) on the classification accuracy. Supplementary Fig. 8a shows the classification accuracies when we did not use the distance and/or angle features. As shown in the results, the classification accuracies do not change significantly even when we include the distance and angle features. This may be because the trained DNNs could learn concepts related to these features only from the speed and relative angular speed or these additional features are not useful for the classification. However, in many cases, using both the distance and angle features improves classification accuracies.

In addition, we investigate the effect of the original longitude and latitude values on the classification accuracy for the analysis of wild animals (seabirds and bears). Supplementary Fig. 8b shows the classification accuracies when we did not use the longitude and latitude values as DNN inputs. As shown in the results for the seabirds, the latitude and longitude values slightly improve the classification accuracy because the female seabirds prefer a specific place. As for the results of the bears, the latitude and longitude values improve the classification accuracy by approximately 20%. Because the data set contains multiple trajectories (50-day trajectories) from each individual bear and the main habitation area of each bear is different, the

latitude and longitude values improve the accuracy. In our study, we did not use the latitude and longitude values to capture sex-specific characteristics.

Comparison with classic approaches

Here, to show the usefulness of DeepHL by comparing it with classic approaches, we performed a decision tree analysis on the trajectory data from the six animal species (using Scikit-learn v0.21.2) because the analysis can provide interpretable rules. We first extracted the statistical features (mean, standard deviation, variance, maximum, minimum, median, length, and sum using tsfresh v0.12.0) from each time-series of a primitive feature used in our analysis (i.e., speed, angular speed, and distances and angle from initial position) for each trajectory, then performed a decision tree analysis, i.e., building a binary classification decision tree that classifies a feature vector extracted from each trajectory into an appropriate class. Unlike our approach based on LSTM and convolutional layers, this classic approach cannot capture the temporal dynamics of the locomotion data.

Supplementary Fig. 7a-f shows the shallow nodes in a tree for each animal study, and Supplementary Fig. 7g summarizes the classification accuracies for DeepHL and the decision trees to indicate the distinguishing power of the classifiers. The root node of each tree shows the most distinguishing feature, i.e., feature with the highest information gain. The tree for worms indicates that the variance of the distance from the initial position is the most distinguishing feature. Although it is convincing that the movement distance of preexposed worms is long, this fact is not novel [12]. (The preexposed worms efficiently escape from odor.) Unlike when using DeepHL, obtaining the temporal dynamics of locomotion (e.g., variance of speed in a certain state) by using the classic approach is difficult. Here, remember that DeepHL has a function to provide the difference among the distributions of each handcrafted feature of the two classes within highlighted segments. Fig. 1d in the main text was created using the function, indicating the difference of the moving variance of speed calculated for each time slice between the preexposed and control worms within highlighted segments. We can also create a similar graph by using entire trajectories, as shown in Supplementary Fig. 7h. Note that this graph is created without using the outputs of DeepHL (i.e., highlighted segments). We can also determine the difference in the moving variance of speed between the preexposed and control worms. However, this result does not indicate that the feature is actually useful for classification of trajectories because the feature is computed for each time slice. (For example, it is possible that some outlying trajectories can distort the histogram.) In addition, the feature importance of the variance of speed calculated for each entire trajectory is low and is not used in the shallow nodes in the decision tree. Therefore, we can state that the finding obtained by using DeepHL cannot be obtained only from Supplementary Fig. 7h without the information provided by DeepHL.

As for the trees for mice and beetles, speed-related features are the most distinguishing features. Although it is convincing that speed-related features are useful in distinguishing between PD and normal animals, this fact is also not novel [48].

Similar to DeepHL, the tree for crickets indicates that the rotation-related feature is important. However, this classic approach does not provide information about temporal dynamics (i.e., which portions of trajectories are important). Although the knowledge extracted from the tree for seabirds (importance of the longitude-related feature) is similar to that obtained by using DeepHL, it is difficult to realize the females' preference for coastlines without highlighted trajectories provided by DeepHL. As for the tree for bears, the speed-related feature is the most distinguishing feature. This result relates to the fact revealed by using DeepHL, but DeepHL enabled us to reveal more specific locomotion characteristics (movements between location clusters).

As above, the classic approach provides analysis based only on high-level features prepared in advance. In contrast, DeepHL enables us to design new high-level features based on trained DeepHL-Net. Specifically, the attention mechanism permits us to focus on important trajectory segments, facilitating designing high-level features that capture temporal dynamics.

Herein, we also introduce another prior study [49] mainly performed by specialists who have been intensively studying the behavior of worms based on a classic approach on the worm data that are also used in our study. Briefly speaking, the method proposed in the study extracts handcrafted features within each trajectory segment and calculates the information gain for each feature to identify a distinguishing feature. Although the method comprehensively extracted the 333 locomotion features, it could not find such dynamic locomotion features discovered by DeepHL because such features are beyond the scope of the worm researchers' assumption.

Animals

Worm. Young adult wild-type hermaphrodite *C. elegans* were used in this study. The techniques used for culturing and handling *C. elegans* were performed as described previously (Brenner, 1974) [50]. The *C. elegans* wild-type Bristol strain were obtained from the Caenorhabditis Genetics Center (University of Minnesota, USA) and cultivated in 6 cm nematode growth medium agar plates with a lawn of *Escherichia coli* strain OP50.

Mouse. The mice were C57BL/6J males and females purchased from Shimizu Laboratory Supplies (Kyoto, Japan) and 6–17 months old at time of testing. Animals were group-housed at 23 °C, with food and water provided ad libitum in a 12h light and 12h dark cycle (day starting at 09:00). All tests were performed during the light period.

Beetle. The *Tribolium castaneum* beetle culture used in this study has been maintained in laboratories for more than 25 years. The beetles were fed wholemeal (Yoshikura Shokai, Tokyo, Japan) enriched with brewer's yeast (Asahi Beer, Tokyo, Japan) as the rearing medium and kept in a chamber (Sanyo, Tokyo, Japan) maintained at 25 °C and 60% RH under a photoperiod

of 16:8 h light:dark cycle (lights on at 07:00, light off at 23:00). The strains with short (S-strain) and long (L-strain) duration of tonic immobility were used. The number of the S-strain (L-strain) beetles is 20, consisting of 10 males and 10 females.

Cricket. Laboratory-bred adult male crickets (*Gryllus bimaculatus* De Geer) (0.50–0.80 g body weight) within 2 weeks after the imaginal molt were used. They were reared under 12 h light:12 h dark conditions at a constant temperature of 27°C. All crickets were tested individually, were naive, and picked at random from the plastic container within which they were reared. We removed their antennae to eliminate the influence of mechanosensory inputs from the antennal organ so we could focus on the interaction between the cercal and auditory systems.

Seabird. Male and female streaked shearwaters (*Calonectris leucomelas*) living on Awashima Island (38° 28'N, 139° 14'E; Niigata, Japan) were used (male: 70, female: 78). The sex of the birds was determined based on their vocalizations during handling; males give high-pitched calls, whereas females give low-pitched calls (Arima et al. 2014)[51]. The age of each bird was unknown. GPS loggers (GiPSy-2, $37 \times 16 \times 4$ mm or GiPSy-4, $37 \times 19 \times 6$ mm; TechnoSmArt, Roma, Italy) were attached to the back feathers of chick-rearing streaked shearwaters with Tesa tape (Beiersdorf AG; GmbH, Hamburg, Germany) and cyanoacrylate glue (Loctite 401; Henkel Ltd., Hatfield, UK). The loggers were housed in waterproof heat-shrink tubing and the total weight of the unit was 25 g.

Bear. Asian black bears (*Ursus thibetanus*) captured in the Ashio-Nikko Mountains areas, in the central part of Honshu Island, Japan (approximately 460 km^2; 36°54'–36°80'E, 139°22'–139°49'N) were used. Between 2006 and 2015, 36 Asian black bears were captured with barrel traps baited with honey. The trapped bears were immobilized with a Tiletamine hydrochloride and Zolazepam hydrochloride mixture (8 mg/kg estimated body mass; Zoletil; Virbac, Carros, France). The sex of each bear was determined and the upper first premolar tooth was extracted for age determination (male: 18, female: 18). Bears were equipped with GPS collars (Lotek GPS3300S and GPS4400S; Lotek, Ontario, Canada, Followit AB; Followit, Lindesberg, Sweden, and GPS Plus and GPS Plus Iridium; Vectronic aerospace, Berlin, Germany).

Statistics. We used the lmerTest package (v. 2.0–36) of R (v. 3.4.3) for the GLMM analysis of the seabirds, crickets, and bears.

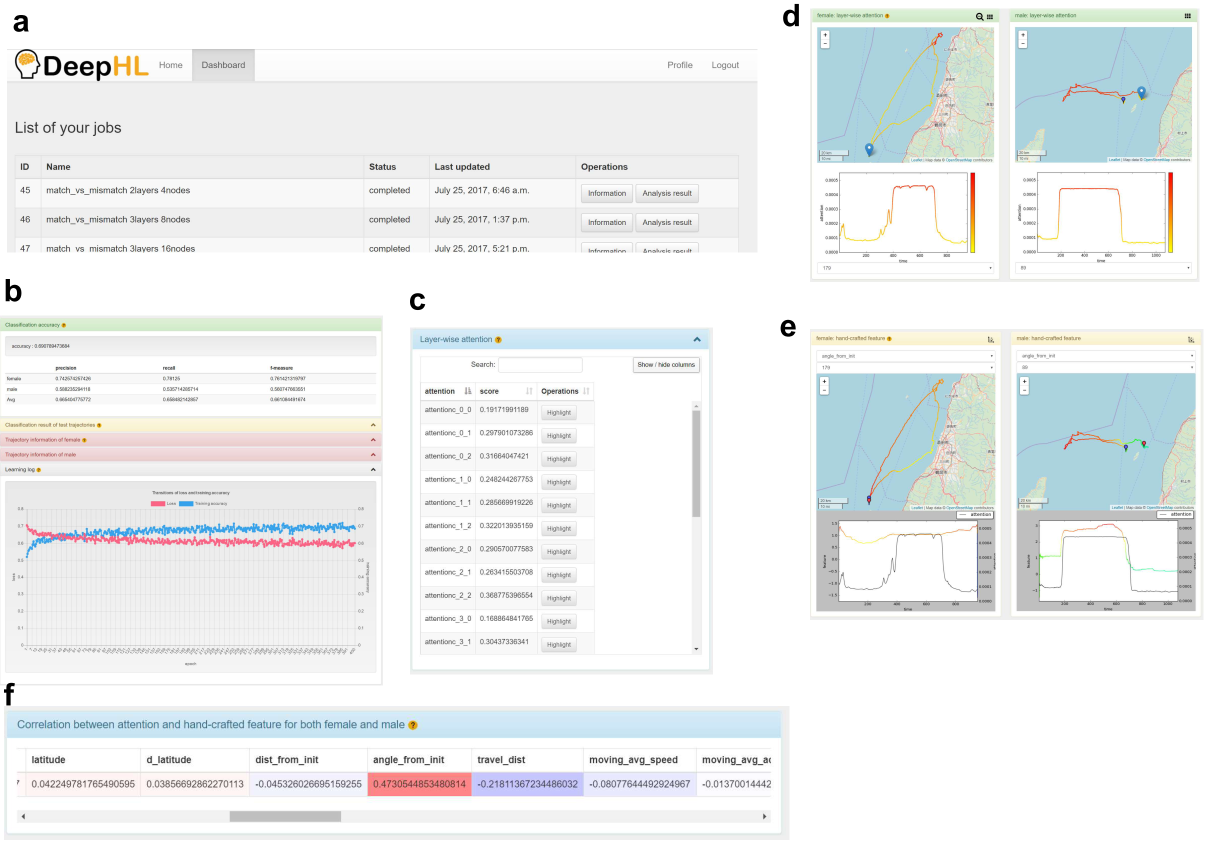

Supplementary Figure 1. Screenshots of the DeepHL web interface. **a** Dashboard page. **b** Analysis result page providing information about the classification performance of trained DeepHL-Net and the transitions of loss and accuracy during training. **c** Analysis result page providing a list of scores of convolutional/LSTM layers. A user can sort the layers according to the scores. **d** Highlighting result page showing trajectories highlighted by attention of a selected layer. **e** Highlighting result page showing trajectories colored by a handcrafted feature or sensor data. **f** Highlighting result page showing a list of correlation coefficients between the attention values of the selected layer and values of each of handcrafted features or sensor data. Base map and data copyright OpenStreetMap contributors (License: www.openstreetmap.org/copyright).

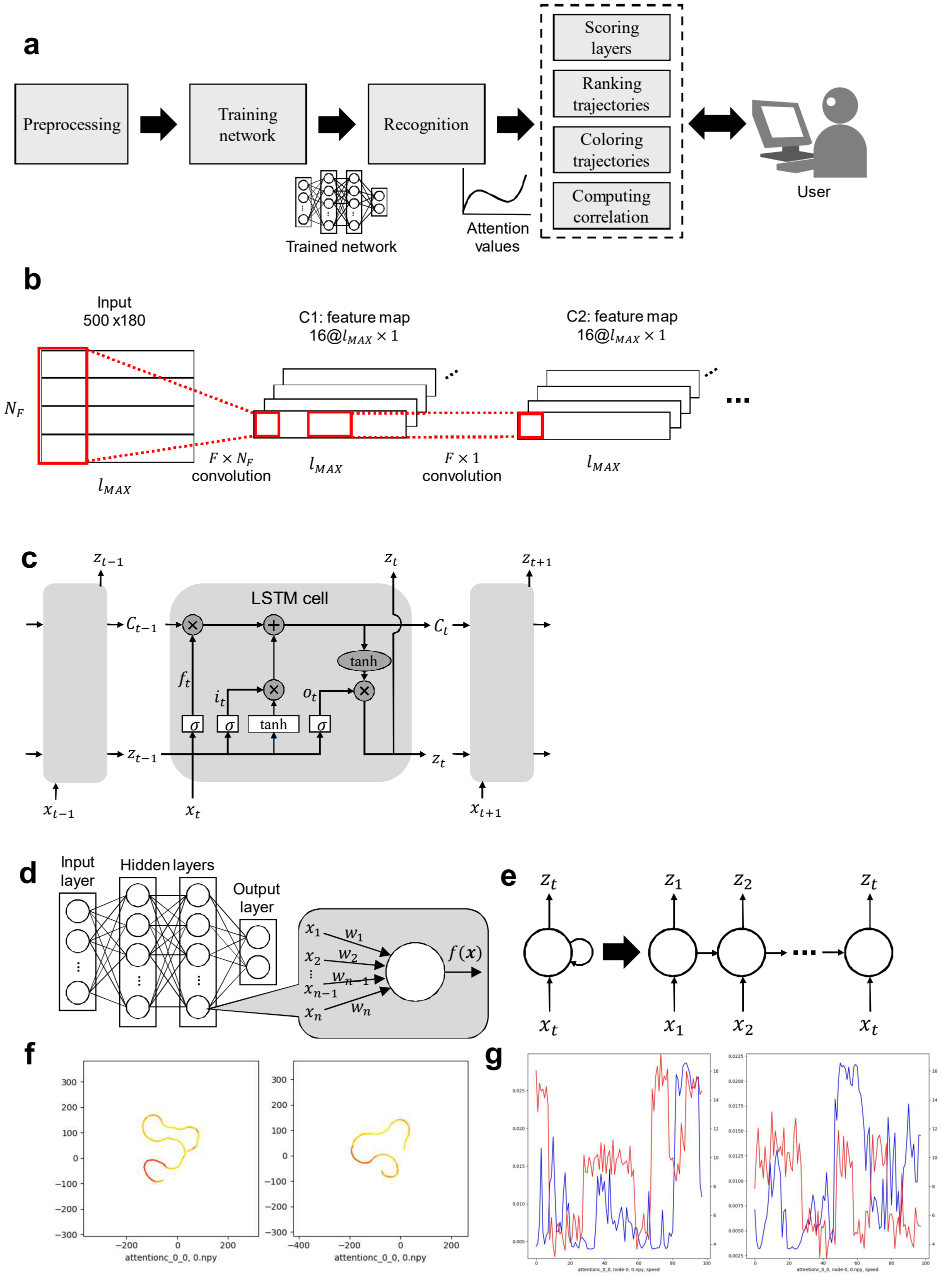

Supplementary Figure 2. Algorithm of DeepHL. **a** Overview of the DeepHL method. **b** Architecture of the CNN layers used in this study. **c** LSTM cell. **d** Typical structure of a feedforward neural network. **e** Simple architecture of an RNN and its unfolded network. **f, g** Screenshots of the user interface of a Python program for DeepHL.

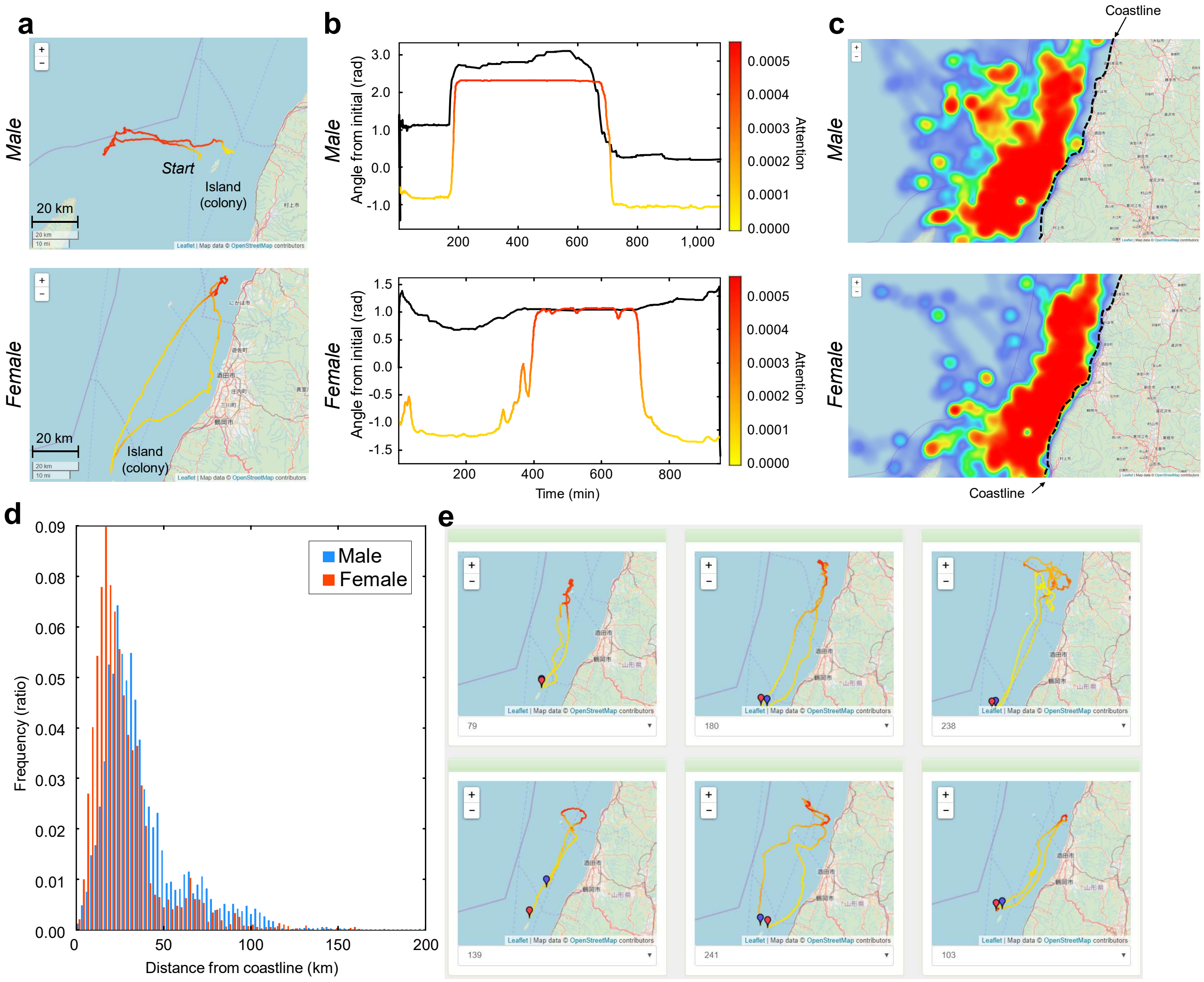

Supplementary Figure 3. DeepHL analysis of the seabirds. **a** Example trajectories of seabirds colored by attention of a discriminator layer. The upper one is the trajectory of a male seabird and the lower one is the trajectory of a female seabird. The male trajectory is highlighted when the male bird travels directly away from the sea coast. The female trajectory shows that segments close to the sea coast are highlighted. **b** Time-series of the angle from the initial position (black lines) associated with attention values (colored lines). The upper and lower graphs are obtained from the upper and lower trajectories shown in (a), respectively. **c** Heatmaps of the GPS measurements of all male and female seabirds. This result shows that the GPS measurements of the female seabirds are closer to the coastline than those of the male seabirds. **d** Distributions of distances between GPS measurements and coastline. A significant difference between male and female seabirds was observed by the GLMMs with Gaussian distributions ($t = 6.068$; df $= 515.5$; $p = 2.51 \times 10^{-9}$, effect size$(r^2) = 0.759$). The p-value is two sided. Individual factors were treated as random effects. The number of data points for the male class is 364,308 and that for the female class is 313,337. We used the lmerTest package (v. 2.0-36) of R (v. 3.4.3). **e** Trajectories of other female birds colored by the discriminator layer. Base map and data copyright OpenStreetMap contributors (License: www.openstreetmap.org/copyright).

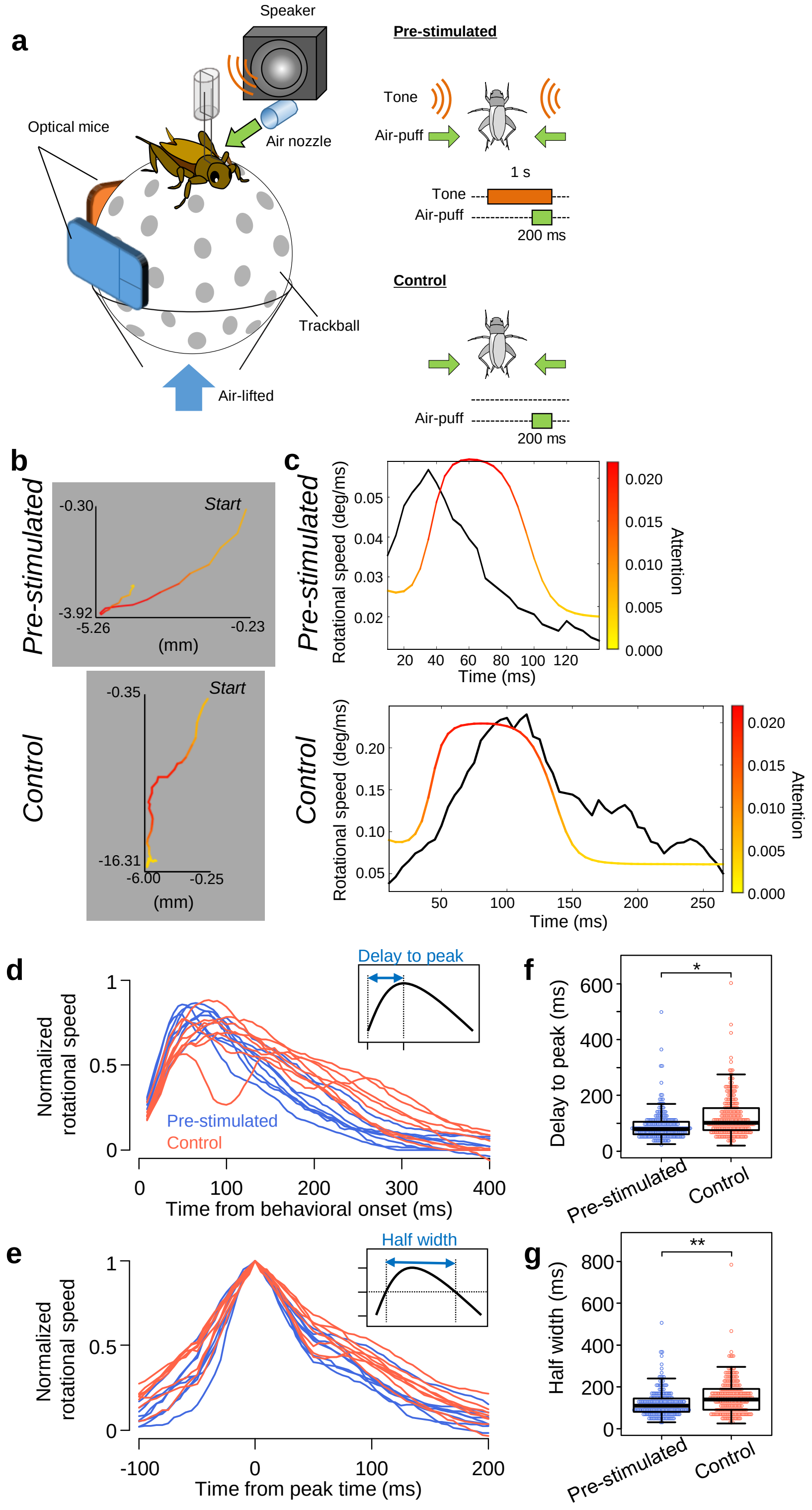

Supplementary Figure 4. DeepHL analysis of the auditory modulation in cricket escape behavior. **a** Experimental apparatus and stimulation protocols. For the with-tone group, a 10-kHz tone sound of 1-s duration was presented 800 ms before an air-puff for 200 ms. For the without-tone group, the air-puff was delivered without any sound. **b** Example trajectories colored with a discriminator layer. **c** Time-series of rotational speed (black lines) and attention value (colored lines) of the trajectories in (b). The rotational speed was filtered using a moving average. **d, e** Time-series of the rotational speed normalized to the maximum value in each trajectory. Each trace indicates the averaged time-series for each individual ($N = 8$ for with-tone; $N = 8$ for without-tone). Data were aligned in the temporal axis with the behavioral onset (d) or with the time of peak value (e) before calculating the average. Insets show the definitions of delay-to-peak and half-width metrics. **f, g** Pooled data of the delay-to-peak (f) and half width (g) features. The box plot whiskers indicate the $1.5\times$ interquartile range of the lower and upper quartiles; box limits indicate the lower, median, and upper quartiles from bottom to top. Each dot shows data for a single trial of a trajectory ($n = 206$ trajectories from eight individual with-tone crickets; $n = 240$ trajectories from eight individual without-tone crickets). Delay to peak: $t = 2.523$; $df = 14.493$; $p = .0239$, effect size$(r^2) = 0.180$, half width: $t = 3.124$; df $= 15.254$; $p = .00685$, effect size$(r^2) = 0.088$ (GLMM with Gaussian distributions; $*p < 0.05, **p < 0.01$). All p-values are two sided.

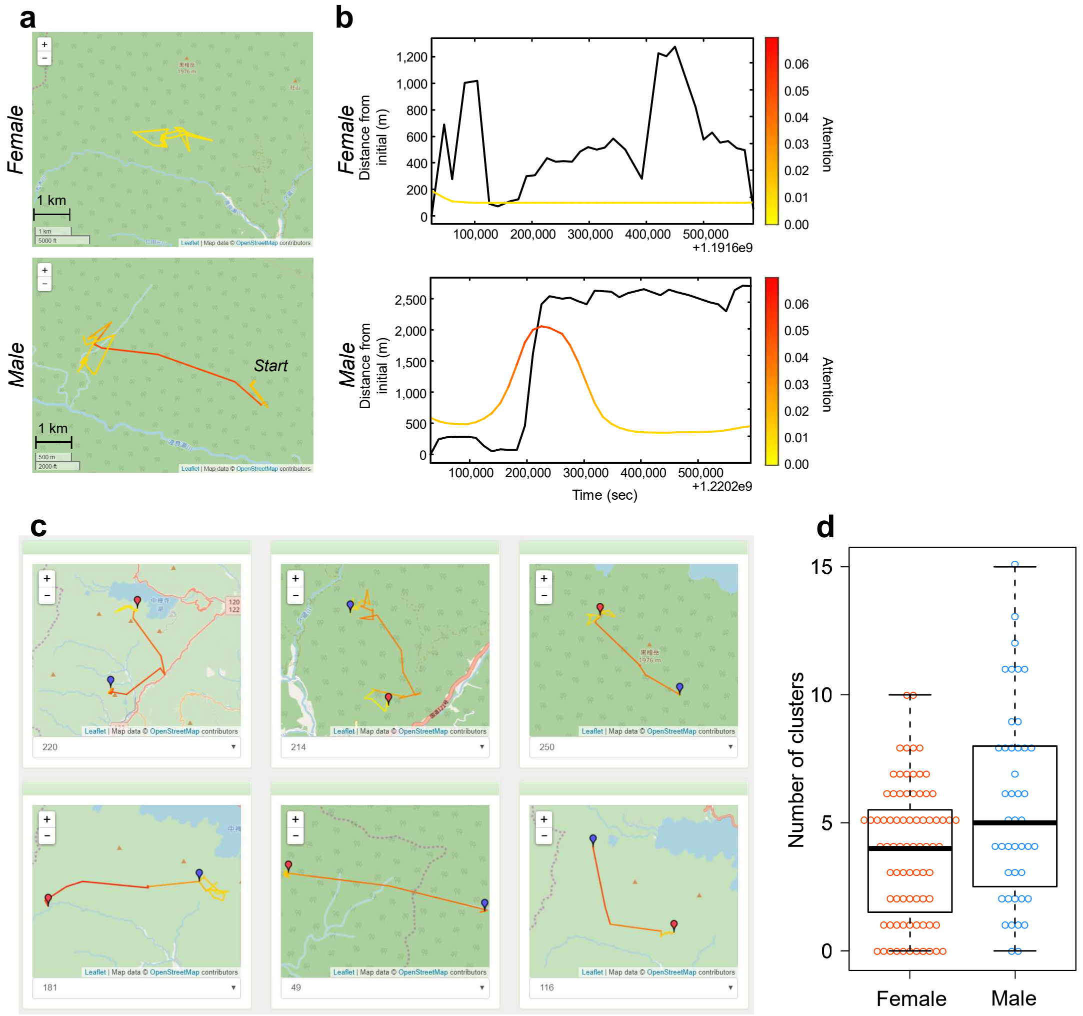

Supplementary Figure 5. DeepHL analysis of the bears. **a** Example trajectories of female and male bears colored by the attention values of a discriminator layer. The upper one is a trajectory of the female bear and the lower one is a trajectory of the male bear. The lower trajectory shows that segments corresponding to long distance travel before/after long-term stays are highlighted. **b** Time-series of the travel distance from the initial position (black lines) associated with attention values (colored lines). The upper and lower graphs are obtained from the upper and lower trajectories shown in (a), respectively. The lower graph shows that attention values have large positive values when the distance increases. **c** Other male trajectories colored by attention of the discriminator layer. **d** Distributions of the numbers of clusters extracted from 50-day GPS data. A significant difference between the male and female classes was observed by GLMMs with Gaussian distributions ($t = 2.435$; df $= 26.9$; $p = 0.0218$, effect size$(r^2) = 0.447$). The p-value is two sided. Individual factors were treated as random effects. $n = 44$ for male and $n = 79$ for female. We used the lmerTest package (v. 2.0-36) of R (v. 3.4.3). The box plot shows the 25–5% quartile, with embedded bar representing the median; whiskers show the minimum and maximum values. Dots show values for individual sessions. Base map and data copyright OpenStreetMap contributors (License: www.openstreetmap.org/copyright).

a

	1st state	2nd state	3rd state
Mean of speed (m/s)	5	10	15
Std. of speed (m/s)	1	1	1
Mean of angular speed (rad/s)	(±)0.1	(±)0.1	(±)0.1
Std. of angular speed (rad/s)	0.1	0.1	0.2

b

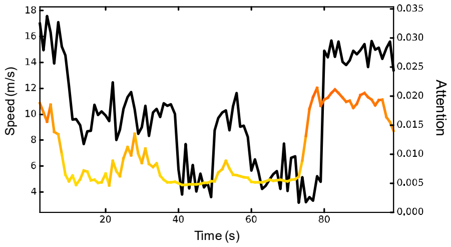

c

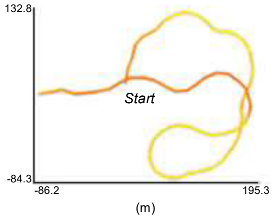

d

	Precision	Recall	F-measure
1st class	0.91	0.68	0.78
2nd class	0.53	0.84	0.65
**Micro avg.**	0.73	0.73	0.73
**Macro avg.**	0.72	0.76	0.71

Supplementary Figure 6. Investigation using synthetic data. **a** Means and standard deviations of normal distributions for movement speed and angular speed prepared for each latent state. The third state is only for class B. **b** Time-series of speed (black lines) associated with the attention values (colored lines) of a trajectory of class B. Segments with speeds of around 15 m/s correspond to the third latent state, which is characteristic of class B. **c** Synthetic trajectory of class B. The discriminator layer pays attention to the red segments. **d** Classification performance for data points of class-B trajectories.

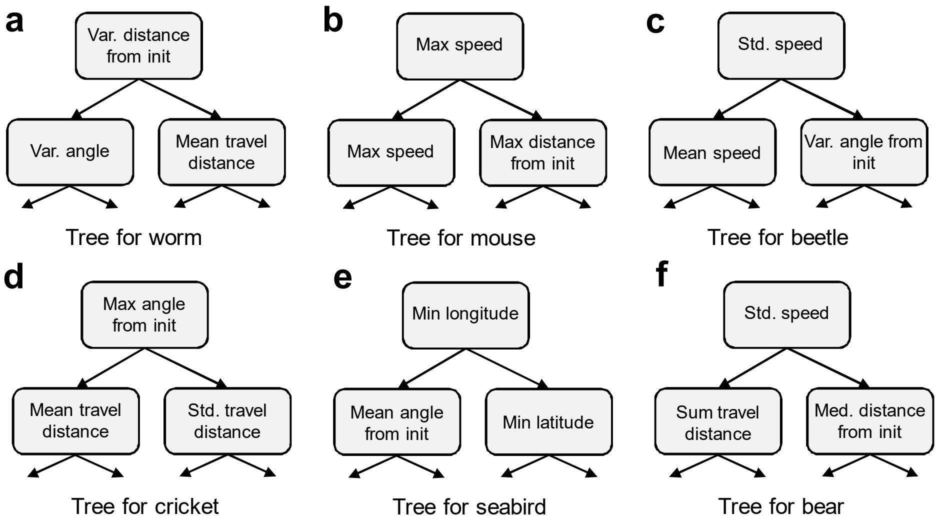

g

	Worm	Mouse	Beetle	Cricket	Seabird	Bear
DeepHL	93.9	74.7	84.5	73.6	65.7	59.8
Decision tree	84.8	70.1	82.1	64.8	61.4	50.0

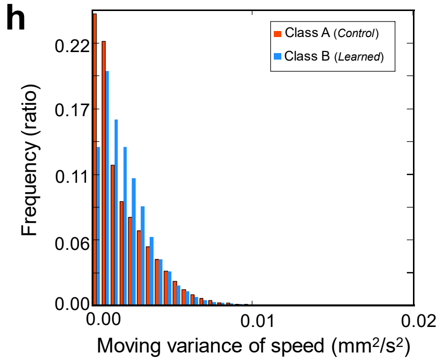

Supplementary Figure 7. Analysis results of the decision tree-based classic approach. **a-f** Decision trees of the animal analysis. **g** Binary classification accuracies (%) for DeepHL and decision trees. **h** Histograms showing the distributions of the moving variance of speed for each time slice computed from entire trajectories of worms.

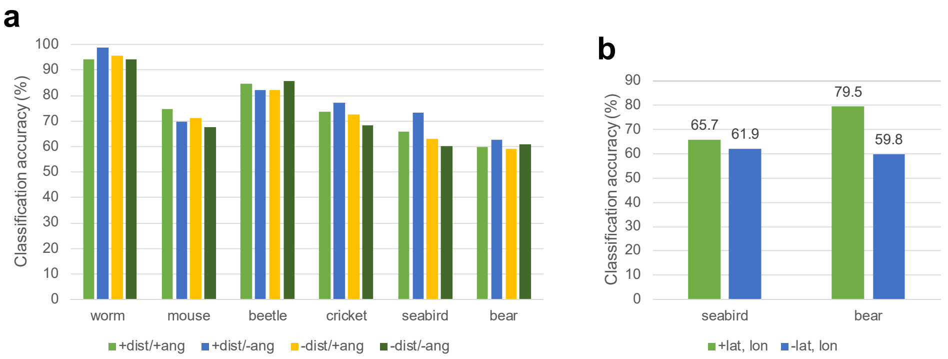

Supplementary Figure 8. Effects of additional features on classification performance: **a** The classification accuracies when we use or do not use additional features, i.e., distances from the initial position and/or angle from the initial position, as DNN inputs. For example, "+dist/-ang" shows a case where the distance features are used but the angle feature is not used as input features. **b** The classification accuracies when we use or do not use the original latitude and longitude as DNN inputs. Here "+lat, lon" shows a case where we use the latitude and longitude information and "-lat, lon" shows a case where we do not use the latitude and longitude information.

Supplementary Table 1. List of handcrafted features. DeepHL automatically computes the correlation coefficient between activation time-series of each artificial neuron and each of the features.

feature	description
speed	Speed based on time unit
acceleration	Acceleration based on time unit
angle	Angle between y-axis and movement direction
relative angular speed	Difference in angle per time unit between current and previous time
angle of acceleration	Y-axial component of acceleration
x	X-coordinate
delta x	Derivative of x
y	Y-coordinate
delta y	Derivative of y
delta of *	Derivative is also calculated for each of sensor data and handcrafted features included in data files.
travel distance from initial	Travel distance between initial and current positions
straight-line distance from initial	Straight-line distance between initial and current positions
angle from initial	Angle between y-axis and straight line connecting initial and current positions
moving average of *	Moving average of each of the above features. Window size is 10. Moving average is also calculated for each of sensor data and handcrafted features included in a data file.
moving variance of *	Moving variance of each of the above features. Window size is 10. Moving variance is also calculated for each of sensor data and handcrafted features included in a data file.

Supplementary Table 2. Overview of the data sets of the six animals used in the deep learning-based analysis

animal	1st category	2nd category	# trajectories (1st)	# trajectories (2nd)	# individuals (1st)	# individuals (2nd)
worm (*Caenorhabditis elegans*)	with learning	without learning	162	163	162	163
cricket (*Gryllus bimaculatus*)	with-tone	without-tone	206	240	8	8
mouse (*Mus musculus*)	normal	PD	374	592	5	4
streaked shearwater (*Calonectris leucomelas*)	male	female	219	205	70	78
red flour beetle (*Tribolium castaneum*)	L-strain	S-strain	230	189	10	10
Asian black bear (*Ursus thibetanus*)	male	female	276	276	18 (14 for statistical test)	18

animal	sampling rate	avg. duration of trajectories	avg. data length (data points)	additional sensor data	data collection
worm (*Caenorhabditis elegans*)	1 Hz	9.9 m	596	n/a	camera
cricket (*Gryllus bimaculatus*)	200 Hz	267.2 s	52	body-axis angle, body-axis angular speed, integral of body-axis angle	treadmill
mouse (*Mus musculus*)	62.4 Hz	30.0 s	1873	n/a	camera
streaked shearwater (*Calonectris leucomelas*)	0.016 Hz	16.0 h	928	n/a	GPS
red flour beetle (*Tribolium castaneum*)	14.3 Hz	52.0 s	984	n/a	treadmill
Asian black bear (*Ursus thibetanus*)	1 sample per 2 h	153.5 h	36	n/a	GPS

Supplementary Table 3. Highly correlated features with attention values of discriminator layers of interest

	feature	coefficient	feature	coefficient	feature	coefficient
animal	worm		cricket		mouse	
1st	moving avg. of speed	0.451	moving avg. of derivative of x	-0.391	distance from init	0.232
2nd	moving avg. of derivative of y	-0.303	moving avg. of body-axis angular speed	-0.352	travel distance	0.148
3rd	speed	0.285	body-axis angular speed	-0.327	moving var. of y	0.124
4th	moving var. of angular speed	-0.278	x	-0.269	moving var. of x	-0.119
5th	derivative of y	-0.237	moving avg. of x	-0.268	moving avg. of speed	0.078

	feature	coefficient	feature	coefficient	feature	coefficient
animal	sea bird		beetle		bear	
1st	angle from init	-0.473	travel distance	-0.121	moving avg. of speed	0.078
2nd	travel distance	-0.218	distance from init	-0.106	distance from init	0.069
3rd	moving avg. of angle	0.131	moving var of acceleration of angle	-0.081	moving var. of derivative of latitude	0.058
4th	angle	0.089	moving avg. of speed	-0.077	moving var. of derivative of latitude	0.056
5th	moving avg. of speed	-0.081	moving avg. of acceleration	-0.064	moving var. of derivative of y	0.056

Supplementary Table 4. Information that a user inputs when he/she adds a new job

Items	Descriptions
Name of job	-
Description of job	-
Longitude and latitude information (optional)	Indices of columns containing longitude and latitude information in trajectory data files
Additional DNN inputs (optional)	Indices of columns containing additional DNN inputs such as sensor data and handcrafted features in trajectory data files
Using distance features as DNN inputs	Whether or not travel distance and airline distance from the start point are used as DNN inputs
Using angle feature as DNN input	Whether or not angle from start point are used as DNN inputs
Number of layers	Number of LSTM layers included in DeepHL-Net (Default: 2)
Number of nodes	Number of artificial neurons included in each layer (Default: 8)
Number of epochs	Number of epochs of DNN training (Default: 300)

Supplementary Table 5. Effects of the amount of data on classification performance when using the worm data. (80% of trajectories are used as training data.) Generally speaking, using sufficient data yields high performance. However, we could confirm that using only 80 trajectories still achieved high accuracy.

# trajectories	classification accuracy	correctly classified	wrongly classified
325	0.939	62	4
160	0.941	32	2
80	0.889	16	2
40	0.800	8	2

Supplementary References

1. Vlachos, M., Gunopulos, D. & Das, G. Rotation invariant distance measures for trajectories. In *2004 ACM SIGKDD International Conference on Knowledge Discovery and Data Mining*, 707–712 (2004). URL http://portal.acm.org/citation.cfm?doid=1014052.1014144.
2. Bengio, Y., Simard, P. & Frasconi, P. Learning long-term dependencies with gradient descent is difficult. *IEEE Transactions on Neural Networks* **5**, 157–166 (1994). arXiv:1211.5063v2.
3. Gers, F. A., Schmidhuber, J. & Cummins, F. Learning to forget: Continual prediction with LSTM. *Neural Computation* **12**, 2451–2471 (2000). arXiv:1011.1669v3.
4. Graves, A., Mohamed, A.-R. & Hinton, G. Speech recognition with deep recurrent neural networks. In *2013 IEEE International Conference on Acoustics, Speech and Signal Processing (ICASSP)*, 6, 6645–6649 (2013). arXiv:1303.5778v1.
5. Ordóñez, F. J. & Roggen, D. Deep convolutional and LSTM recurrent neural networks for multimodal wearable activity recognition. *Sensors* **16**, 115 (2016).
6. Kingma, D. P. & Ba, J. L. Adam: a method for stochastic optimization. *arXiv preprint arXiv:1412.6980* 1–15 (2014). 1412.6980v9.
7. Rumelhart, D. E., Hinton, G. E. & Williams, R. J. Learning representations by back-propagating errors. *Nature* **323**, 533–536 (1986). arXiv:1011.1669v3.
8. Werbos, P. J. Backpropagation through time: What it does and how to do it. *Proceedings of the IEEE* **78**, 1550–1560 (1990).
9. Baek, J. H., Cosman, P., Feng, Z., Silver, J. & Schafer, W. R. Using machine vision to analyze and classify *Caenorhabditis elegans* behavioral phenotypes quantitatively. *Journal of Neuroscience Methods* **118**, 9–21 (2002).
10. Stephens, G. J., Johnson-Kerner, B., Bialek, W. & Ryu, W. S. Dimensionality and dynamics in the behavior of *C. elegans*. *PLoS Computational Biology* **4**, e1000028 (2008). 0705.1548.
11. Brown, A. E. X., Yemini, E. I., Grundy, L. J., Jucikas, T. & Schafer, W. R. A dictionary of behavioral motifs reveals clusters of genes affecting *Caenorhabditis elegans* locomotion. *Proceedings of the National Academy of Sciences* **110**, 791–796 (2013).
12. Kimura, K. D., Fujita, K. & Katsura, I. Enhancement of odor avoidance regulated by dopamine signaling in *Caenorhabditis elegans*. *The Journal of neuroscience : the official journal of the Society for Neuroscience* **30**, 16365–16375 (2010).
13. Yamazaki, S. J. *et al.* Experience-dependent modulation of behavioral features in sensory navigation of nematodes and bats revealed by machine learning. *bioRxiv 198879* (2017). 198879.
14. Miyatake, T., Nakayama, S., Nishi, Y. & Nakajima, S. Tonically immobilized selfish prey can survive by sacrificing others. *Proceedings of the Royal Society B: Biological Sciences* **276**, 2763–2767 (2009).
15. Rogers, S. M. & Simpson, S. J. Thanatosis. *Current Biology* **24**, R1031–R1033 (2014).
16. Humphreys, R. K. & Ruxton, G. D. A review of thanatosis (death feigning) as an anti-predator behaviour. *Behavioral Ecology and Sociobiology* **72**, 22 (2018).
17. Miyatake, T. *et al.* Is death-feigning adaptive? Heritable variation in fitness difference of death-feigning behaviour. *Proceedings of the Royal Society B: Biological Sciences* **271**, 2293–2296 (2004).
18. Miyatake, T. *et al.* Pleiotropic antipredator strategies, fleeing and feigning death, correlated with dopamine levels in *Tribolium castaneum*. *Animal Behaviour* **75**, 113–121 (2008).
19. Tribolium Genome Sequencing Consortium. The genome of the model beetle and pest *Tribolium castaneum*. *Nature* **452**, 949–55 (2008).
20. Darwin, C. *The Descent of Man and Selection in Relation to Sex*, vol. 1 (John Murray, 1888). arXiv:1011.1669v3.
21. Kays, R., Crofoot, M. C., Jetz, W. & Wikelski, M. Terrestrial animal tracking as an eye on life and planet. *Science* **348**, aaa2478 (2015). /doi.org/10.1126/science.aaa2478.
22. Hussey, N. E. *et al.* Aquatic animal telemetry: A panoramic window into the underwater world. *Science* **348**, 1255642 (2015).
23. Weimerskirch, H., Louzao, M., De Grissac, S. & Delord, K. Changes in wind pattern alter albatross distribution and life-history traits. *Science* **335**, 211–214 (2012).
24. Yamamoto, T. *et al.* Geographical variation in body size of a pelagic seabird, the streaked shearwater *Calonectris leucomelas*. *Journal of Biogeography* **43**, 801–808 (2016).
25. Matsumoto, S., Yamamoto, T., Yamamoto, M., Zavalaga, C. B. & Yoda, K. Sex-related differences in the foraging movement of streaked shearwaters *Calonectris leucomelas* breeding on Awashima Island in the Sea of Japan. *Ornithological Science* **16**, 23–32 (2017).
26. Suryan, R. M. *et al.* Wind, waves, and wing loading: Morphological specialization may limit range expansion of endangered albatrosses. *PLoS ONE* **3**, e4016 (2008).
27. Navarro, J. & González-Solís, J. Environmental determinants of foraging strategies in Cory's shearwaters *Calonectris diomedea*. *Marine Ecology Progress Series* **378**, 259–267 (2009).
28. Shaffer, S. A., Weimerskirch, H. & Costa, D. P. Functional significance of sexual dimorphism in Wandering Albatrosses, *Diomedea exulans*. *Functional Ecology* **15**, 203–210 (2001).
29. Navarro, J. & González-Solís, J. Experimental increase of flying costs in a pelagic seabird: Effects on foraging strategies, nutritional state and chick condition. *Oecologia* **151**, 150–160 (2007).
30. Freed, L. A. Loss of mass in breeding wrens: Stress or adaptation? *Ecology* **62**, 1179–1186 (1981).

31. Phillips, R. A., Silk, J. R., Phalan, B., Catry, P. & Croxall, J. P. Seasonal sexual segregation in two Thalassarche albatross species: Competitive exclusion, reproductive role specializaion or foraging niche divergence? *Proceedings of the Royal Society B: Biological Sciences* **271**, 1283–1291 (2004).
32. Sakuma, T. *et al.* Finding discriminative animal behaviors from sequential bio-logging trajectory data. In *6th International Conference on Distributed, Ambient and Pervasive Interactions (DAPI 2018)*, 125–138 (2018).
33. Domenici, P. Context-dependent variability in the components of fish escape response: Integrating locomotor performance and behavior. *Journal of Experimental Zoology Part A: Ecological Genetics and Physiology* **313 A**, 59–79 (2010).
34. Fukutomi, M. & Ogawa, H. Crickets alter wind-elicited escape strategies depending on acoustic context. *Scientific Reports* **7**, 15158 (2017).
35. Fukutomi, M., Someya, M. & Ogawa, H. Auditory modulation of wind-elicited walking behavior in the cricket *Gryllus bimaculatus*. *Journal of Experimental Biology* **218**, 3968–3977 (2015).
36. Marler, P. The mountain gorilla: Ecology and behavior. *Science* **140**, 1081–1082 (1963).
37. Hebblewhite, M. & Haydon, D. T. Distinguishing technology from biology: A critical review of the use of GPS telemetry data in ecology. *Philosophical Transactions of the Royal Society B: Biological Sciences* **365**, 2303–2312 (2010).
38. Koike, S. Long-term trends in food habits of the Asiatic black bear in the Misaka Mountains, Japan. *Mammal. Biol.* **75(1)**, 17–28 (2010).
39. Koike, S. *et al.* Effect of hard mast production on foraging and sex-specific behavior of the Asiatic black bear (*Ursus thibetanus*). *Mammal Study* **37**, 21–28 (2012).
40. Kozakai, C. *et al.* Effect of mast production on home range use of Japanese black bears. *Journal of Wildlife Management* **75**, 867–875 (2011).
41. Kozakai, C. *et al.* Fluctuation of daily activity time budgets of Japanese black bears: relationship to sex, reproductive status, and hard-mast availability. *J. Mammal.* **94(2)**, 351–360 (2013).
42. Izumiyama, S., Mochizuki, T., Kishimoto, R., Gotoh, M. & Hayashi, H. Elucidation of massive haunt factor to Asiatic black bear depend to rural area capture time and age assessment in the Nagano Prefecture. *Bulletin of the Shinshu University Alpine Field Center* **6**, 19–24 (2008).
43. Ester, M., Kriegel, H.-P., Sander, J. & Xu, X. A density-based algorithm for discovering clusters in large spatial databases with noise. In *International Conference on Knowledge Discovery and Data Mining*, 226–231 (1996).
44. Macqueen, J. Some methods for classification and analysis of multivariate observations. In *Fifth Berkeley Symposium on Mathematical Statistics and Probability*, vol. 1, 281–297 (1967).
45. Hazumi, T. & Maruyama, N. Movements and home ranges of Japanese black bears in Nikko. *Bears: Their Biology and Management* **6**, 99–101 (1986).
46. Ohnishi, N. & Osawa, T. A difference in the genetic distribution pattern between the sexes in the Asian black bear. *Mammal Study* **39**, 11–16 (2014).
47. Kozakai, C. *et al.* Influence of food availability on matrilineal site fidelity of female Asian black bears. *Mammal Study* **42**, 219–230 (2017).
48. Kravitz, A. V. *et al.* Regulation of parkinsonian motor behaviors by optogenetic control of basal ganglia circuitry. *Nature* **466**, 622–626 (2013).
49. Yamazaki, S. J. *et al.* STEFTR: A hybrid versatile method for state estimation and feature extraction from the trajectory of animal behavior. *Frontiers in Neuroscience* **13**, 626 (2019).
50. Brenner, S. The genetics of *Caenorhabditis elegans*. *Genetics* **77**, 71–94 (1974). arXiv:1408.1149.
51. Arima, H., Oka, N., Baba, Y., Sugawa, H. & Ota, T. Gender identification by calls and body size of the streaked shearwater examined by CHD genes. *Ornithological Science* **13**, 9–17 (2014).
