# Supplementary material for: Deep learning-assisted comparative analysis of animal trajectories with DeepHL
Source: Nat Commun. 2020 Oct 20;11:5316. doi: 10.1038/s41467-020-19105-0 (PMC7576204; doi:10.1038/s41467-020-19105-0)
Supplement: Supplementary file 3 — Description of Additional Supplementary Files [file 41467_2020_19105_MOESM3_ESM.pdf]

Description of Additional Supplementary Files

File Name: Supplementary Data 1
Description: Datasets analysed in this study

File Name: Supplementary Movie 1
Description: Browsing highlighted trajectories of seabirds. Base map and data from OpenStreetMap and OpenStreetMap Foundation (License: openstreetmap.org).

File Name: Supplementary Software 1
Description: Python program of DeepHL
