# Supplementary material for: Deep learning-assisted comparative analysis of animal trajectories with DeepHL
Source: Nat Commun. 2020 Oct 20;11:5316. doi: 10.1038/s41467-020-19105-0 (PMC7576204; doi:10.1038/s41467-020-19105-0)
Supplement: Supplementary file 7 — Reporting Summary [file 41467_2020_19105_MOESM7_ESM.pdf]

nature research

Corresponding author(s): Takuya Maekawa
NCOMMS-19-386264

Last updated by author(s): Sep 15, 2020

Reporting Summary

Nature Research wishes to improve the reproducibility of the work that we publish. This form provides structure for consistency and transparency in reporting. For further information on Nature Research policies, see Authors & Referees and the Editorial Policy Checklist.

Statistics

For all statistical analyses, confirm that the following items are present in the figure legend, table legend, main text, or Methods section.

n/a	Confirmed	
☐	☒	The exact sample size (*n*) for each experimental group/condition, given as a discrete number and unit of measurement
☐	☒	A statement on whether measurements were taken from distinct samples or whether the same sample was measured repeatedly
☐	☒	The statistical test(s) used AND whether they are one- or two-sided<br>*Only common tests should be described solely by name; describe more complex techniques in the Methods section.*
☒	☐	A description of all covariates tested
☒	☐	A description of any assumptions or corrections, such as tests of normality and adjustment for multiple comparisons
☐	☒	A full description of the statistical parameters including central tendency (e.g. means) or other basic estimates (e.g. regression coefficient) AND variation (e.g. standard deviation) or associated estimates of uncertainty (e.g. confidence intervals)
☐	☒	For null hypothesis testing, the test statistic (e.g. *F*, *t*, *r*) with confidence intervals, effect sizes, degrees of freedom and *P* value noted<br>*Give P values as exact values whenever suitable.*
☒	☐	For Bayesian analysis, information on the choice of priors and Markov chain Monte Carlo settings
☒	☐	For hierarchical and complex designs, identification of the appropriate level for tests and full reporting of outcomes
☐	☒	Estimates of effect sizes (e.g. Cohen's *d*, Pearson's *r*), indicating how they were calculated

Our web collection on statistics for biologists contains articles on many of the points above.

Software and code

Policy information about availability of computer code

Data collection

[worm]
Move-tr/2D (Library Inc., Japan; v. 8.31)

[cricket]
Tracktaro (Chinou Jouhou Shisutemu Inc., Japan; v. 1.0.4)
Hardware: Custom made treadmill system

[mouse]
Custom softwares based on Matlab (R2018b, Mathworks, Ma, USA) and LabVIEW(Labview 2018, National Instruments, TX, USA) were used for tracking.
Hardware: Dragonfly Express (IEEE1394 digital camera; Point Grey Research, BC, Canada) , PCIe-8255R (Reconfigurable digital I/O Frame Grabber; National Instruments, TX, USA)

[seabird]
No software was used.
Hardware: GiPSy-2, 37×16×4 mm or GiPSy-4, 37×19×6 mm; TechnoSmArt, Roma, Italy

[beetle]
Custom software based on OpenCV (https://opencv.org/; v. 2.4.9) was used for tracking.
Hardware: ANTAM (see Nagaya et al. 2017 PLOS ONE, doi.org/10.1371/journal.pone.0177480)

[bear]
No software was used.
Hardware: Lotek GPS3300S and GPS4400S; Lotek, Ontario, Canada, Followit AB; Followit, Lindesberg, Sweden, and GPS Plus and GPS Plus Iridium; Vectronic aerospace, Berlin, Germany

Data analysis

<Statistical tests>
[worm]
R v. 3.4.3
lmerTest package v. 2.0-36

[cricket]
R v. 3.4.3
lmerTest package v. 2.0-36

[mouse]
R v. 3.2.3
exactRankTests package v. 0.8-29

[seabird]
R v. 3.4.3
lmerTest package v. 2.0-36

[beetle]
JMP 12.2.0., SAS

[bear]
R v. 3.4.3
lmerTest package v. 2.0-36

<Analysis using deep learning>
Anaconda v4.5.11, CUDA v8.0.0, CuDNN v7.0.5, Python v3.6.6, Tensorflow (gpu): v1.4.1, Keras (gpu): v2.0.8

For manuscripts utilizing custom algorithms or software that are central to the research but not yet described in published literature, software must be made available to editors/reviewers. We strongly encourage code deposition in a community repository (e.g. GitHub). See the Nature Research guidelines for submitting code & software for further information.

Data

Policy information about availability of data

All manuscripts must include a data availability statement. This statement should provide the following information, where applicable:

- Accession codes, unique identifiers, or web links for publicly available datasets
- A list of figures that have associated raw data
- A description of any restrictions on data availability

The dataset of the worms analysed during the current study is available in the Dryad repository, https://doi.org/10.5061/dryad.37pvmcvf5, and included in the supplementary information file. The datasets of the mice, beetles, crickets, and seabirds analysed during the current study are included in the supplementary information file. The dataset of the bears are available from the corresponding author upon reasonable request because the release of the bear data can increase the likelihood of poaching and stir up the fear in residents.

Field-specific reporting

Please select the one below that is the best fit for your research. If you are not sure, read the appropriate sections before making your selection.

☒ Life sciences ☐ Behavioural & social sciences ☐ Ecological, evolutionary & environmental sciences

For a reference copy of the document with all sections, see nature.com/documents/nr-reporting-summary-flat.pdf

Life sciences study design

All studies must disclose on these points even when the disclosure is negative.

Sample size

[worm]
A large scale behavioral analysis of C. elegans concluded that 20 worms would discriminate single SD in a behavioral phenotype at over 80% power (Yemini et al., 2013). We used 50-100 worms per condition to investigate multiple aspects of behavior in detail.

[cricket]
We used 16 male crickets (with tone: N=8, without tone: N=8) in the present study. This sample size was determined by the time available to perform experiments combined with cricket availability.

[mouse]
We used 10 mice (wild type: N=5, PD model: N=5) in the present study. Sample size was chosen based on effect strength of similar experiments (Kravitz et al., Nature 466, pp. 622–626 (2010)) for PD.

[seabird]
Sample size was determined by the time available and the availability of GPS sensor data loggers.
Hundreds of foraging trips are needed to estimate home range correctly (Soanes et al., 2013); therefore, we used 70 males and 78 females in the present study, in each of which birds has 3-4 foraging trips on average.

[beetle]
We used 40 beetles (male: N =20, female: N = 20) in the present study. This sample size was determined by the time available to perform experiments combined with beetle availability.

[bear]
Sample size was determined by the time available and the availability of GPS sensor data loggers. Because a prior study identified the bears' differences in home ranges and behavior patterns between summer and fall using 13 bears (Koike et al., 2012), we used 18 males and 18 females in the present study.

Data exclusions

[worm]
Since worms do not exhibit odor avoidance behavior during the first two minutes because of rapid increase in odor concentration (Tanimoto 2017), the data for the following 10 minutes (i.e. 600 s) was used.

[cricket]
We excluded the data of animals that did not respond to the air-puff stimulus or started to move during 800-ms period before the air puff. The classification criteria of the animals' responses have been established in the previous study (Fukutomi et al., 2015, doi:10.1242/jeb.128751).

[mouse]
We excluded 30-second segments that contain no movements of a mouse for deep learning-based analysis. This is because the purpose of this study is to find locomotion features of PD/healthy mice. In contrast, 10-minute trajectories were not excluded in the analysis of the three behavioral features.

[seabird]
We used only data collected during a foraging trip, which is defined as the time a bird spent beyond a 3-km buffer zone around the colony. In addition, we discarded data of short trips (< 700 data samples). When trajectories with different lengths are processed in deep learning, zero-padding is used so that all the trajectories have a uniform length. Because short trajectories are composed mostly of zero values, we discarded short trajectories. Because the long trajectories have the length of approximately 2,500, we selected about 30% of the length as the threshold.

[beetle]
No data was excluded.

[bear]
To avoid potential bias due to capture, data collected in the 48 hours following release from capture were excluded. In addition, we discarded 1-week travel data with small numbers of GPS measurements (< 30). When trajectories with different lengths are processed in deep learning, zero-padding is used so that all the trajectories have a uniform length. Because trajectories with many missing values are composed mostly of zero values, we discarded these trajectories. Because complete trajectories have the length of approximately 90, we selected about 30% of the length as the threshold.

Replication

[worm]
Experiments were repeated for 4 days or more per condition to verify reproduceabitity.

[cricket]
For each individual cricket, we measured the trajectory from four sessions of experiments, each of which comprised 10 trials, were performed using the same protocol. In other words, we repeatedly obtained the data of 40 trials in total from each individual. We confirmed no interaction of between the auditory effects and the experimental session (Fukutomi et al., 2015, doi:10.1242/jeb.128751). This means successful replication of the measurements.

[mouse]
The measurements were repeated at least twice to verify reproducibility.

[seabird]
We did not repeat the measurements. For an ethical reason, we did not deploy data loggers on the birds that were used before.

[beetle]
We have collected trajectories of 20 individual beetles from each strain. All experiments were conducted with single individuals. All repeated experiments were successful.

[bear]
We did not repeat the measurements because it is difficult to capture the same bear again.

Randomization

[worm]
The order of experimental condition was randomized on a daily basis.

[cricket]
The measurements were not randomized because the same type of stimulation protocol was applied to each individual cricket.

[mouse]
Individual mice with different sexes and ages are randomly used for measurement.

[seabird]
We selected individual birds from the breeding colony at random by assuming that they were representative of the population.

[beetle]
The measurements were randomized on a daily basis.

[bear]
The measurements were performed for bears that we could capture in the field by assuming that they were randomly-selected representative of the population.

Blinding

[worm]
Blinding was not required because all the data collection and analysis were performed automatically.

[cricket]
We did not consider blinding of the measurements. All the data collection and analysis were performed automatically.

[mouse]
We did not consider blinding of the measurements. All the data collection and analysis were performed automatically.

[seabird]
We did not consider blinding of the measurements. Blinding was not relevant to our study.

[beetle]
We did not consider blinding of the measurements, but the data collection was performed randomly, and we did not tell the experimenters what the treatment and control zones meant, so it was essentially a blind experiment.

[bear]
We did not consider blinding of the measurements. Blinding was not relevant to our study.

Behavioural & social sciences study design

All studies must disclose on these points even when the disclosure is negative.

Study description

Briefly describe the study type including whether data are quantitative, qualitative, or mixed-methods (e.g. qualitative cross-sectional, quantitative experimental, mixed-methods case study).

Research sample

State the research sample (e.g. Harvard university undergraduates, villagers in rural India) and provide relevant demographic information (e.g. age, sex) and indicate whether the sample is representative. Provide a rationale for the study sample chosen. For studies involving existing datasets, please describe the dataset and source.

Sampling strategy

Describe the sampling procedure (e.g. random, snowball, stratified, convenience). Describe the statistical methods that were used to predetermine sample size OR if no sample-size calculation was performed, describe how sample sizes were chosen and provide a rationale for why these sample sizes are sufficient. For qualitative data, please indicate whether data saturation was considered, and what criteria were used to decide that no further sampling was needed.

Data collection

Provide details about the data collection procedure, including the instruments or devices used to record the data (e.g. pen and paper, computer, eye tracker, video or audio equipment) whether anyone was present besides the participant(s) and the researcher, and whether the researcher was blind to experimental condition and/or the study hypothesis during data collection.

Timing

Indicate the start and stop dates of data collection. If there is a gap between collection periods, state the dates for each sample cohort.

Data exclusions

If no data were excluded from the analyses, state so OR if data were excluded, provide the exact number of exclusions and the rationale behind them, indicating whether exclusion criteria were pre-established.

Non-participation

State how many participants dropped out/declined participation and the reason(s) given OR provide response rate OR state that no participants dropped out/declined participation.

Randomization

If participants were not allocated into experimental groups, state so OR describe how participants were allocated to groups, and if allocation was not random, describe how covariates were controlled.

Ecological, evolutionary & environmental sciences study design

All studies must disclose on these points even when the disclosure is negative.

Study description

Briefly describe the study. For quantitative data include treatment factors and interactions, design structure (e.g. factorial, nested, hierarchical), nature and number of experimental units and replicates.

Research sample

Describe the research sample (e.g. a group of tagged Passer domesticus, all Stenocereus thurberi within Organ Pipe Cactus National Monument), and provide a rationale for the sample choice. When relevant, describe the organism taxa, source, sex, age range and any manipulations. State what population the sample is meant to represent when applicable. For studies involving existing datasets, describe the data and its source.

Sampling strategy	*Note the sampling procedure. Describe the statistical methods that were used to predetermine sample size OR if no sample-size calculation was performed, describe how sample sizes were chosen and provide a rationale for why these sample sizes are sufficient.*
Data collection	*Describe the data collection procedure, including who recorded the data and how.*
Timing and spatial scale	*Indicate the start and stop dates of data collection, noting the frequency and periodicity of sampling and providing a rationale for these choices. If there is a gap between collection periods, state the dates for each sample cohort. Specify the spatial scale from which the data are taken*
Data exclusions	*If no data were excluded from the analyses, state so OR if data were excluded, describe the exclusions and the rationale behind them, indicating whether exclusion criteria were pre-established.*
Reproducibility	*Describe the measures taken to verify the reproducibility of experimental findings. For each experiment, note whether any attempts to repeat the experiment failed OR state that all attempts to repeat the experiment were successful.*
Randomization	*Describe how samples/organisms/participants were allocated into groups. If allocation was not random, describe how covariates were controlled. If this is not relevant to your study, explain why.*
Blinding	*Describe the extent of blinding used during data acquisition and analysis. If blinding was not possible, describe why OR explain why blinding was not relevant to your study.*

Did the study involve field work? ☐ Yes ☐ No

Field work, collection and transport

Field conditions	*Describe the study conditions for field work, providing relevant parameters (e.g. temperature, rainfall).*
Location	*State the location of the sampling or experiment, providing relevant parameters (e.g. latitude and longitude, elevation, water depth).*
Access and import/export	*Describe the efforts you have made to access habitats and to collect and import/export your samples in a responsible manner and in compliance with local, national and international laws, noting any permits that were obtained (give the name of the issuing authority, the date of issue, and any identifying information).*
Disturbance	*Describe any disturbance caused by the study and how it was minimized.*

Reporting for specific materials, systems and methods

We require information from authors about some types of materials, experimental systems and methods used in many studies. Here, indicate whether each material, system or method listed is relevant to your study. If you are not sure if a list item applies to your research, read the appropriate section before selecting a response.

Materials & experimental systems

n/a	Involved in the study	
☐	☒	Antibodies
☒	☐	Eukaryotic cell lines
☒	☐	Palaeontology
☐	☒	Animals and other organisms
☒	☐	Human research participants
☒	☐	Clinical data

Methods

n/a	Involved in the study	
☒	☐	ChIP-seq
☒	☐	Flow cytometry
☒	☐	MRI-based neuroimaging

Antibodies

Antibodies used	Mouse:<br>For immunostaining, sections were divided into six interleaved sets. Immunohistochemistry was performed on the free floating sections. Sections were pretreated with 3% hydrogen peroxide and incubated overnight with primary antibody mouse anti-tyrosine hydroxylase (1:1000; Millipore). As a secondary antibody, we used biotinylated donkey anti-mouse IgG (1:100; Jackson ImmunoResearch Inc.) followed by incubation with avidin-biotin-peroxydase complex solution (1:100; VECTASTAIN Elite ABC STANDARD KIT, Vector labo-ratories).
Validation	Mouse:<br>All antibodies (primary antibody: mouse anti-tyrosine hydroxylase (MAB318, Millipore), secondary antibody: biotinylated donkey anti-mouse IgG (AB_2340785, Jackson ImmunoResearch Inc.)) have been validated by the manufacturers.

Eukaryotic cell lines

Policy information about cell lines

Cell line source(s)	*State the source of each cell line used.*
Authentication	*Describe the authentication procedures for each cell line used OR declare that none of the cell lines used were authenticated.*
Mycoplasma contamination	*Confirm that all cell lines tested negative for mycoplasma contamination OR describe the results of the testing for mycoplasma contamination OR declare that the cell lines were not tested for mycoplasma contamination.*
Commonly misidentified lines (See ICLAC register)	*Name any commonly misidentified cell lines used in the study and provide a rationale for their use.*

Palaeontology

Specimen provenance	*Provide provenance information for specimens and describe permits that were obtained for the work (including the name of the issuing authority, the date of issue, and any identifying information).*
Specimen deposition	*Indicate where the specimens have been deposited to permit free access by other researchers.*
Dating methods	*If new dates are provided, describe how they were obtained (e.g. collection, storage, sample pretreatment and measurement), where they were obtained (i.e. lab name), the calibration program and the protocol for quality assurance OR state that no new dates are provided.*

☐ Tick this box to confirm that the raw and calibrated dates are available in the paper or in Supplementary Information.

Animals and other organisms

Policy information about studies involving animals; ARRIVE guidelines recommended for reporting animal research

Laboratory animals

[worm]
Young adult state wild-type hermaphrodite C. elegans were used in this study. The techniques used for culturing and handling C. elegans were performed as described previously (Brenner, 1974). The C. elegans wild-type Bristol strain were obtained from the Caenorhabditis Genetics Center (University of Minnesota, USA) and cultivated in 6 cm nematode growth medium (NGM) agar plates with a lawn of Escherichia coli strain OP50.

[cricket]
Laboratory-bred adult male crickets (Gryllus bimaculatus De Geer) (0.50–0.80 g body weight) within 2 weeks after the imaginal molt were used. Most crickets molt as adults at 7 to 8 weeks after hatching, so the crickets used were approximately 7 to 10 weeks old. They were reared under 12 h light:12 h dark conditions at a constant temperature of 27°C. All crickets were tested individually, were naive and picked at random from a plastic container within which they were reared. We removed their antennae to eliminate the influence of mechanosensory inputs from the antennal organ so we could focus on the interaction between the cercal and auditory systems.

[mouse]
Mice were C57BL/6J males and females purchased from Shimizu laboratory supplies (Kyoto, Japan) and 6 - 17 months old at time of testing. Animals were group-housed at room temperature of 20-26°C and humidity of 40-60%, with food and water provided ad libitum in a 12h light and 12h dark cycle (day starting at 9:00 a.m.). All tests were performed during the light period.

[beetle]
The Tribolium castaneum beetle culture used in this study has been maintained in laboratories for more than 25 years. The beetles were fed wholemeal (Yoshikura Shokai, Tokyo, Japan) enriched with brewer's yeast (Asahi Beer, Tokyo, Japan) as the rearing medium and kept in a chamber (Sanyo, Tokyo, Japan) maintained at 25°C and 60% RH under a photoperiod of 16:8 h light:dark cycle (lights on at 0700, light off at 2300). The strains with short (S-strain) and long (L-strain) duration of tonic immobility were used. The number of the S-strain (L-strain) beetles is 20, consisting of 10 males and 10 females. The age of beetles used were about 20 d old.

Wild animals

[seabird]
Male and female streaked shearwaters (Calonectris leucomelas) living in Awashima Island (38°28′N, 139°14′E; Niigata, Japan) were used (male: 70, female: 78). Seabirds were captured from their nest burrows by hand. The sex of the birds was determined based on their vocalizations during handling; males give high-pitched calls, whereas females give low-pitched calls (Arima et al. 2014). The age of each bird was unknown. GPS loggers (GiPSy-2, 37×16×4 mm or GiPSy-4, 37×19×6 mm; TechnoSmArt, Roma, Italy) were attached to the back feathers of chick-rearing streaked shearwaters at the colony with Tesa tape (Beiersdorf AG; GmbH, Hamburg, Germany) and cyanoacrylate glue (Loctite 401; Henkel Ltd., Hatfield, UK). The loggers were housed in waterproof heat-shrink tubing and the total weight of the unit was 25 g. We released the seabirds at the colony just after retrieving the loggers.

[bear]
Asian black bears (Ursus thibetanus) captured in the Ashio-Nikko Mountains areas, in the central part of Honshu Island, Japan (approximately 460 km2; E36.54–36.80, N139.22–139.49) were used. Between 2006 and 2015, 36 Asian black bears were captured with barrel traps baited with honey. The trapped bears were immobilized with a Tiletamine hydrochloride and

	Zolazepam hydrochloride mixture (8 mg/kg estimated body mass; Zoletil; Virbac, Carros, France). The sex of each bear is determined (male: 18, female: 18) and extracted upper first premolar tooth for age determination (male avg. age: 7.78, female avg. age: 6,28 at the time of capture). Bears were equipped with GPS collars (Lotek GPS3300S and GPS4400S; Lotek, Ontario, Canada, Followit AB; Followit, Lindesberg, Sweden, and GPS Plus and GPS Plus Iridium; Vectronic aerospace, Berlin, Germany). Immobilized bears were released at the trap sites. Because the GPS collar automatically drops off after about a year, the survival of the bear after it drops off is unknown. It is unknown for bears that died naturally, but at least most of these bears have not been culled after these tracking. Bear capture and handling methods were performed in accordance with the guidelines for animal research established by the Mammal Society of Japan.
Field-collected samples	No field collected samples were used in this study.
Ethics oversight	The studies on streaked shearwaters, mice, and bears were approved by the Animal Experimental Committees of Nagoya University (streaked shearwaters), the Doshisha University Institutional Animal Care and Use Committees (mice), and the Institutional Animal Care and Use Committee of Tokyo University of Agriculture and Technology (bears), respectively. The research on streaked shearwaters was conducted with permits from the Ministry of the Environment, Japan. All experimental procedures used in the bear research followed the Guidelines Concerning Animal Experimentation of the Tokyo University of Agriculture and Technology and the Mammal Society of Japan. They specify no requirements for the treatment of insects in experiments.

Note that full information on the approval of the study protocol must also be provided in the manuscript.

Human research participants

Policy information about studies involving human research participants

Population characteristics	*Describe the covariate-relevant population characteristics of the human research participants (e.g. age, gender, genotypic information, past and current diagnosis and treatment categories). If you filled out the behavioural & social sciences study design questions and have nothing to add here, write "See above."*
Recruitment	*Describe how participants were recruited. Outline any potential self-selection bias or other biases that may be present and how these are likely to impact results.*
Ethics oversight	*Identify the organization(s) that approved the study protocol.*

Note that full information on the approval of the study protocol must also be provided in the manuscript.

Clinical data

Policy information about clinical studies

All manuscripts should comply with the ICMJE guidelines for publication of clinical research and a completed CONSORT checklist must be included with all submissions.

Clinical trial registration	*Provide the trial registration number from ClinicalTrials.gov or an equivalent agency.*
Study protocol	*Note where the full trial protocol can be accessed OR if not available, explain why.*
Data collection	*Describe the settings and locales of data collection, noting the time periods of recruitment and data collection.*
Outcomes	*Describe how you pre-defined primary and secondary outcome measures and how you assessed these measures.*

ChIP-seq

Data deposition

☐ Confirm that both raw and final processed data have been deposited in a public database such as GEO.

☐ Confirm that you have deposited or provided access to graph files (e.g. BED files) for the called peaks.

Data access links<br>*May remain private before publication.*	*For "Initial submission" or "Revised version" documents, provide reviewer access links. For your "Final submission" document, provide a link to the deposited data.*
Files in database submission	*Provide a list of all files available in the database submission.*
Genome browser session<br>(e.g. UCSC)	*Provide a link to an anonymized genome browser session for "Initial submission" and "Revised version" documents only, to enable peer review. Write "no longer applicable" for "Final submission" documents.*

Methodology

Replicates	*Describe the experimental replicates, specifying number, type and replicate agreement.*
Sequencing depth	*Describe the sequencing depth for each experiment, providing the total number of reads, uniquely mapped reads, length of reads and whether they were paired- or single-end.*

Antibodies	*Describe the antibodies used for the ChIP-seq experiments; as applicable, provide supplier name, catalog number, clone name, and lot number.*
Peak calling parameters	*Specify the command line program and parameters used for read mapping and peak calling, including the ChIP, control and index files used.*
Data quality	*Describe the methods used to ensure data quality in full detail, including how many peaks are at FDR 5% and above 5-fold enrichment.*
Software	*Describe the software used to collect and analyze the ChIP-seq data. For custom code that has been deposited into a community repository, provide accession details.*

Flow Cytometry

Plots

Confirm that:

- [ ] The axis labels state the marker and fluorochrome used (e.g. CD4-FITC).
- [ ] The axis scales are clearly visible. Include numbers along axes only for bottom left plot of group (a 'group' is an analysis of identical markers).
- [ ] All plots are contour plots with outliers or pseudocolor plots.
- [ ] A numerical value for number of cells or percentage (with statistics) is provided.

Methodology

Sample preparation	*Describe the sample preparation, detailing the biological source of the cells and any tissue processing steps used.*
Instrument	*Identify the instrument used for data collection, specifying make and model number.*
Software	*Describe the software used to collect and analyze the flow cytometry data. For custom code that has been deposited into a community repository, provide accession details.*
Cell population abundance	*Describe the abundance of the relevant cell populations within post-sort fractions, providing details on the purity of the samples and how it was determined.*
Gating strategy	*Describe the gating strategy used for all relevant experiments, specifying the preliminary FSC/SSC gates of the starting cell population, indicating where boundaries between "positive" and "negative" staining cell populations are defined.*

- [ ] Tick this box to confirm that a figure exemplifying the gating strategy is provided in the Supplementary Information.

Magnetic resonance imaging

Experimental design

Design type	*Indicate task or resting state; event-related or block design.*
Design specifications	*Specify the number of blocks, trials or experimental units per session and/or subject, and specify the length of each trial or block (if trials are blocked) and interval between trials.*
Behavioral performance measures	*State number and/or type of variables recorded (e.g. correct button press, response time) and what statistics were used to establish that the subjects were performing the task as expected (e.g. mean, range, and/or standard deviation across subjects).*

Acquisition

Imaging type(s)	*Specify: functional, structural, diffusion, perfusion.*
Field strength	*Specify in Tesla*
Sequence & imaging parameters	*Specify the pulse sequence type (gradient echo, spin echo, etc.), imaging type (EPI, spiral, etc.), field of view, matrix size, slice thickness, orientation and TE/TR/flip angle.*
Area of acquisition	*State whether a whole brain scan was used OR define the area of acquisition, describing how the region was determined.*

Diffusion MRI ☐ Used ☐ Not used

Preprocessing

Preprocessing software

Provide detail on software version and revision number and on specific parameters (model/functions, brain extraction, segmentation, smoothing kernel size, etc.).

Normalization

If data were normalized/standardized, describe the approach(es): specify linear or non-linear and define image types used for transformation OR indicate that data were not normalized and explain rationale for lack of normalization.

Normalization template

Describe the template used for normalization/transformation, specifying subject space or group standardized space (e.g. original Talairach, MNI305, ICBM152) OR indicate that the data were not normalized.

Noise and artifact removal

Describe your procedure(s) for artifact and structured noise removal, specifying motion parameters, tissue signals and physiological signals (heart rate, respiration).

Volume censoring

Define your software and/or method and criteria for volume censoring, and state the extent of such censoring.

Statistical modeling & inference

Model type and settings

Specify type (mass univariate, multivariate, RSA, predictive, etc.) and describe essential details of the model at the first and second levels (e.g. fixed, random or mixed effects; drift or auto-correlation).

Effect(s) tested

Define precise effect in terms of the task or stimulus conditions instead of psychological concepts and indicate whether ANOVA or factorial designs were used.

Specify type of analysis: ☐ Whole brain ☐ ROI-based ☐ Both

Statistic type for inference
(See Eklund et al. 2016)

Specify voxel-wise or cluster-wise and report all relevant parameters for cluster-wise methods.

Correction

Describe the type of correction and how it is obtained for multiple comparisons (e.g. FWE, FDR, permutation or Monte Carlo).

Models & analysis

n/a	Involved in the study
☐	☐ Functional and/or effective connectivity
☐	☐ Graph analysis
☐	☐ Multivariate modeling or predictive analysis

Functional and/or effective connectivity

Report the measures of dependence used and the model details (e.g. Pearson correlation, partial correlation, mutual information).

Graph analysis

Report the dependent variable and connectivity measure, specifying weighted graph or binarized graph, subject- or group-level, and the global and/or node summaries used (e.g. clustering coefficient, efficiency, etc.).

Multivariate modeling and predictive analysis

Specify independent variables, features extraction and dimension reduction, model, training and evaluation metrics.
